# A Micro-Immunotherapy Sequential Medicine MIM-seq Displays Immunomodulatory Effects on Human Macrophages and Anti-Tumor Properties towards In Vitro 2D and 3D Models of Colon Carcinoma and in an In Vivo Subcutaneous Xenograft Colon Carcinoma Model

**DOI:** 10.3390/ijms23116059

**Published:** 2022-05-27

**Authors:** Camille Jacques, Irene Marchesi, Francesco Paolo Fiorentino, Mathias Chatelais, Nicoletta Libera Lilli, Kurt Appel, Beatrice Lejeune, Ilaria Floris

**Affiliations:** 1 Preclinical Research Department, Labo’Life France, 1 rue François Bruneau, 44000 Nantes, France; ilaria.floris@labolife.com; 2 Kitos Biotech s.r.l.s., Porto Conte Ricerche, S.P. 55 Porto Conte—Capo Caccia, Km 8,400 Loc. Tramariglio, 07041 Alghero, Italy; imarchesi@kitosbiotech.org (I.M.); fpfiorentino@kitosbiotech.org (F.P.F.); 3 ProfileHIT, 7 rue du Buisson, 44680 Sainte-Pazanne, France; mathias.chatelais@profile-hit.com; 4 Centre d’Investigation Clinique Hématologie CHU Hôtel Dieu, 44093 Nantes, France; libera.lilli@gmail.com; 5 VivaCell Biotechnology GmbH, 79211 Denzlingen, Germany; kurt.appel@vivacell.de; 6 InFlectis Bioscience, 44200 Nantes, France; beatricelejeune@inflectisbioscience.com

**Keywords:** micro-immunotherapy, MIM-seq, macrophages, immunomodulation, potential anti-tumor agent, colorectal cancer, 3D tumor spheroids

## Abstract

In this study, the immunomodulatory effects of a sequential micro-immunotherapy medicine, referred as MIM-seq, were appraised in human primary M1 and M2 macrophages, in which the secretion of pro-inflammatory cytokines, such as interleukin (IL)-1β, IL-6, IL-12, IL-23, and tumor necrosis factor (TNF)-alpha, was inhibited. In addition, the potential anti-proliferative effects of MIM-seq on tumor cells was assessed in three models of colorectal cancer (CRC): an in vitro two-dimensions (2D) model of HCT-116 cells, an in vitro tri-dimensional (3D) model of spheroids, and an in vivo model of subcutaneous xenografted mice. In these models, MIM-seq displayed anti-proliferative effects when compared with the vehicle. In vivo, the tumor growth was slightly reduced in MIM-seq-treated animals. Moreover, MIM-seq could slightly reduce the growth of our spheroid models, especially under serum-deprivation. When MIM-seq was combined with two well-known anti-cancerogenic agents, either resveratrol or etoposide, MIM-seq could even further reduce the spheroid’s volume, pointing up the need to further assess whether MIM-seq could be beneficial for CRC patients as an adjuvant therapy. Altogether, these data suggest that MIM-seq could have anti-tumor properties against CRC and an immunomodulatory effect towards the mediators of inflammation, whose systemic dysregulation is considered to be a poor prognosis for patients.

## 1. Introduction

Colorectal cancer (CRC) represents the third most commonly diagnosed cancer and the second deadliest [1]. Genetic alterations, such as somatic and/or germline mutations, combined with epigenetic modulations, are recognized as the major events leading to the carcinogenesis of the colonic epithelium. Conventional treatments for CRC are surgery, chemotherapy and radiotherapy, which can be used alone or in combination to increase efficacy against the tumor [2,3]. In spite of their potent and destructive actions against tumors, chemotherapy and radiotherapy both display many side effects, mostly due to their cytotoxicity toward any highly proliferating cells [2,3].

In this context, evolving therapeutic strategies would, thus, ideally aim at limiting collateral cytotoxic effects on healthy cells, while directly targeting the tumor. Current preclinical research confirms, and highlights, the promising and encouraging potential of immunotherapies in cancer management, and points them out as attractive therapeutic modalities to be included amongst anti-cancer treatments available in clinics [4,5,6,7]. Amongst all the different classes of molecules and compounds used in immunotherapy, cytokines are good candidates. These small peptides are mainly secreted by cells belonging to the immune system, but also by endothelial cells, fibroblasts, and stromal cells. These important immune regulators are known for their autocrine, paracrine, and endocrine effects, as they can mediate their actions on (i) the same cytokine-producing cells, (ii) adjacent cells and (iii) distant cells, respectively [8]. These characteristics allow them to play a role in immune system modulation at several levels, as fine-tuners of both immune cell systemic activation and tumor-targeting immune reactions. As a consequence, they can display anti-tumor effects at every phase of the carcinogenesis process, from the tumor’s initiation to its metastatic spreading [5]. In line with these prerequisites, research focused on the use of recombinant cytokines as an immune-therapeutic strategy against cancer has given promising results [9,10]. An increasing number of clinical trials have recently been launched, exploring the safety and efficacy of cytokine-based drugs, employed either as single agents or as multiple cytokines-based drugs, alone or in combination with other immunomodulatory drugs (source www.clinicaltrials.org accessed date: 10 February 2022).

Amongst the cytokines family, the particular role played by interleukins (ILs) in CRC is attracting increasing interest in research, and the anti-tumor properties of the IL-2 family members, IL-2, IL-19 and IL-15, have been recently reported [11]. Regarding the cytokines currently available for use in immunotherapy, human recombinant (hr) interferon (IFN)-alpha (IFN-α) and IL-2 based-drugs are approved by the Food and Drug Administration (FDA) and European Medicines Agency (EMA) for the treatment of several malignancies [9]. However, their high-dose systemic administration is also associated with adverse effects, that could most likely be related to high pleiotropicity of these immune factors, and their involvement in a large number of biological activities [12,13,14]. In the context of cancer, cytokines can: (i) trigger strong acute immune response, accompanied by release of pyrogenic/cytotoxic mediators and additional cytokines [15]; (ii) generate or perpetuate a chronic state of inflammation, that may either initiate oncogenesis, or sustain progression of a tumor already set [16]. As an example, it has been reported that systemic administration of IL-2 at high doses may act as a mitogen, both for CD8^+^ cytotoxic T lymphocytes (CTLs) and for CD4^+^CD25^+^FOXP3^+^ regulatory T cells; therefore, causing antagonistic actions in the context of anti-cancer immunity [17]. For instance, many markers indicating the instauration of chronic systemic inflammation, such as C-reactive protein, IL-6, IL-7, IL-23, and IL-8, were all found to be increased in the serum of patients suffering from CRC [18]. Moreover, as chronic inflammation can contribute to the pathogenesis of CRC and is associated with poor prognosis, its management is also an important parameter to take into account when setting any therapeutic strategy [18,19].

One of the possible approaches to reach beneficial effects while reducing side effects, might be to target multiple pathways at the same time and use lower doses of cytokines. In this regard, Micro-immunotherapy (MI), which uses signaling molecules, such as cytokines, at very low doses in its formulations [20,21,22,23,24,25,26,27,28,29,30], may offer an innovative possibility as an adjuvant therapy in the context of cancer treatment, as it could contribute to improving patient quality of life. MI medicines are impregnated homeopathic sucrose–lactose pillules (also called globules), packed into capsules, intended to be opened and taken every morning in a fasted state. Their oromucosal use can allow optimization of the delivery of the active substances to the lymphoid tissues of the buccal mucosae, and to the immune cells present in the oral cavity [31,32]. One of the specifications of MI medicines resides in their complex formulations of active substances, that can work together in a possible synergistic manner, on different therapeutic targets. In these medicines, not only cytokines, but also growth factors, hormones, neuropeptides and other signaling molecules involved in cell–cell communication under physiological and pathological conditions, are used as bioactive molecules. In addition, nucleic acids are also employed, either as total deoxyribonucleic acid (DNA) and ribonucleic acid, (RNA) (extracted from *Pinus halepensis* and *Foeniculum vulgare*, respectively), or in the form of specific nucleic acids (SNA^®^), designed to target specific genomic or transcript sequences. Altogether, these active ingredients are independently employed in MI medicines at either low doses (LD), to stimulate their own production by the organism, or on the opposite side of the spectrum, at ultra-low doses (ULD), in order to modulate/inhibit their own biological effects.

A specific MI formulation, hereafter referred to as MIM-seq, is the investigational product of this preclinical study. As featured in all of the MI medicines [25,27,28], MIM-seq is a sequential medicine. It thus consists of a sequence of five different capsules containing pillules (MIM-1, MIM-2, MIM-3, MIM-4, and MIM-5), each one containing a unique active substances combination. Consequently, the content of each one of the five capsules that make it up is expected to be taken one after the other, progressively, to provide a unique combination of active substances in chronological order. Concerning the MIM-seq posology, the pillules contained in the first capsule of the entire sequence (MIM-1) should thus be taken on Day 1, the second one (MIM-2) on Day 2 etc.

Considering the inherent complexity of MIM-seq formulation, our approach was to start our investigations with several in vitro studies, focused on only two capsules out of the five (MIM-3 and MIM-4), to investigate their potential effects on immune cells as well as cancer cells. Moreover, one pilot study was also conducted in vivo. Regarding this in vivo part, the entire MIM-seq effect was assessed, as the mice used in our model received the integrality of the medicine, in a sequential manner, reproducing the human intended posology.

Regarding the fact that macrophages are important players involved in the establishment of immune responses occurring in a wide range of pathological conditions, including cancers, an in vitro multi-polarization macrophage model was used in order to investigate the potential effects of MIM-seq from an immune-inflammation standpoint. To assess the potential anti-cancer properties of the medicine, specifically for CRC, human epithelial cell CRC-derived HCT-116 cells were used, as these cells are commonly employed as an in vitro model for CRC biology and drug response studies.

In addition, to evaluate the potential of MIM-seq as an adjuvant treatment for CRC, we investigated its effects when combined together with either a well-known chemotherapeutic agent, etoposide (ETP), or a natural-derived compound displaying promising anti-cancer effects, resveratrol (RSV).

To summarize, this preclinical exploratory study had three main objectives: (i) to investigate the in vitro effect of MIM-3 on the cytokine secretion profile of macrophages cultivated in the presence of three different co-stimulatory factors to mimic specific contexts, including the inflammatory one (ii) to evaluate the potential anti-tumor activities of either the MIM-4 capsule alone, or the entire sequential medicine MIM-seq, in three HCT-116 cells based-models of CRC: two in vitro models (conventional two-dimensions [2D] culture and three-dimensions [3D] spheroids model), and an in vivo model of subcutaneous xenografted mice, (iii) to assess the potential anti-tumor effects of the medicine as adjuvant treatment for CRC, when associated with other anti-carcinogenic agents, such as chemotherapeutic agents, or natural-derived compounds. With this purpose in mind, a pilot experiment was conducted, using ETP and RSV, respectively.

## 2. Results

### 2.1. MIM-3 Acts as an Immune-Modulator of Cytokines’ Secretion in Macrophages Cultivated under Various Immune Contexts

First, in order to investigate the immune-modulatory properties of MIM-3, a model of human CD14^+^-derived macrophages was chosen because of its high plasticity and sensitivity in response to different stimuli [33].

**Figure 1:**
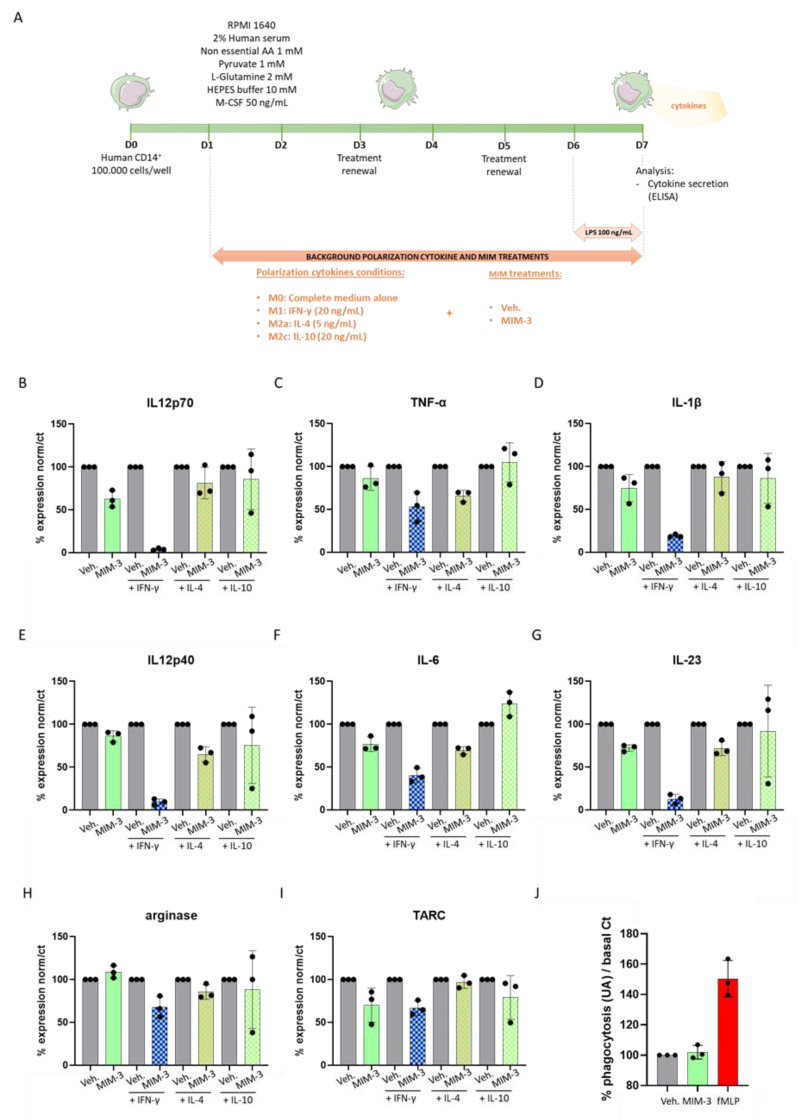
MIM-3 acts as an immune-modulator of macrophages’ cytokine secretion without affecting the phagocytosis capabilities of granulocytes. (**A**). Representative scheme of the CD14^+^-derived macrophages’ cytokine secretion assessment protocol. CD14^+^ cells were obtained from peripheral blood mononuclear cells (PBMCs) isolated from one healthy donor and cultivated for 6 days in complete medium supplemented with M-CSF 50 ng/mL alone (M0), IFN-γ at 20 ng/mL (M1), IL-4 at 5 ng/mL (M2a), or IL-10 at 20 ng/mL (M2c), as background polarization cytokines and in the presence of either MIM-3 or vehicle (Veh.). An additional 24 h of LPS treatment (100 ng/mL) were applied as an inducer of an inflammatory status during the last day of the MIM-3/Veh. treatment. AA: amino acids. Cytokine secretion was evaluated by ELISA. (**B**–**I**) Effect of MIM-3 on the secretion of the cytokines: IL12p70, TNF-α, IL-1β, IL12p40, IL-6, IL-23, arginase and TARC. The results are expressed in percentage of the cytokine secretion in the Veh. -treated conditions, the Veh. being set at 100% for each one of the differentiation backgrounds. The M1-macrophages induced by IFN-γ and treated with MIM-3 are represented with the green- and blue-squared histograms, the M2a macrophages induced by IL-4 and treated with MIM-3 are represented with the green- and pink-squared histograms, and the M2c macrophages induced by IL-10 and treated with MIM-3 are represented with the green- and yellow-squared histograms. Each condition has been performed in triplicate. (**J**) Human granulocytes were preincubated for 10 min with MIM-3, or vehicle (Veh.), or 10 µM N-formyl-methionyl-leucyl-phenylalanine (fMLP) as a positive phagocytosis inducer. Fluorescent beads were then added to the culture medium for an additional 45 min. Each condition was performed in triplicate and each histogram represents the mean ± SD of the fluorescence as a percentage of the Veh.-treated conditions, set at 100%.

The entire protocol is illustrated in Figure 1A. Briefly, after isolation, CD14^+^ cells were cultivated for 24 h in complete basal M0 macrophage conditions. At Day 1 (D1), the cells were supplemented with either 20 ng/mL IFN-γ, 5 ng/mL IL-4 or 20 ng/mL IL-10 as “background cytokines” to induce macrophage polarization towards M1-type, M2a-type or M2c-type, respectively. The concomitant treatment with either the tested MIM-capsule or the vehicle (Veh.) also started at D1. At Day 6 (D6), lipopolysaccharide (LPS) was added to the culture media, at the final working concentration of 100 ng/mL, to induce a pro-inflammatory stimulus, and the cells were harvested at Day 7 (D7) for cytokine secretion analysis by enzyme linked immunosorbent assay (ELISA). The results of the ELISA for the four untreated M0, M1-type, M2a-type or M2c-type macrophages, further exposed to LPS during the last 24 h, are shown in Figure S1. The secretion of IL-12 subunit 70 (IL12p70), TNF-α, IL-1β, IL-12 subunit 40 (IL12p40), IL-6, IL-23, arginase, and thymus and activation-regulated chemokine (TARC) were assessed and the results are presented in Figure 1B–I, as percentages of the Veh. effect in each “background condition”. Overall, MIM-3 decreased the secretion of all cytokines, but not the enzyme arginase, in the basal culture conditions (Figure 1I, plain green histograms). Interestingly enough, these effects on cytokine secretion levels seem to be reinforced in IFN-γ treated cells (Figure 1B–H, green- and blue-squared histograms). Regarding the IL-4 background (i.e., M2a polarization), the basal cytokine secretion of this macrophage subtype also seemed to be modulated by MIM-3 treatment, when compared to the Veh. (Figure 1C–J, green- and pink-squared histograms). Finally, the effects of MIM-3 on cytokine secretion, in the presence of IL-10 (i.e., M2c polarization), appeared to be the slightest (Figure 1C–J, green- and yellow-squared histograms). In this M2c polarization context, as MIM-3 also displayed an overall effect towards a diminution of cytokine secretion, it barely affected TNF-α and stimulated the secretion of IL-6 by about 23%. While additional experiments are needed to confirm the findings, the results presented here suggest that MIM-3 can display an immunomodulatory function towards the reduction of pro-inflammatory cytokine secretion on M0, M1 and M2 macrophages. Altogether, these data suggest that, out of the five capsules of the complete MIM-seq sequence, MIM-3 could participate in the decrease of cancer-related systemic inflammation within an organism, through its action in helping to reduce the release of pro-inflammatory cytokines by macrophages. Transposed into the CRC context, this particular capsule could, thus, act in impeding one of the possible fuels for tumor progression.

Considering the particular composition of the tested MIM capsule, which included IFN-γ (6 CH), IL-12 (4 CH) and IFN-α (3 CH), all cytokines related to phagocytosis promotion [34,35], we wanted to assess if MIM-3 could stimulate immune cell phagocytic capability in a human granulocytes model.

The protocol followed in this experiment was the same as the one previously described by Jacques et al., who highlighted that the MI unitary medicine IFN-γ (4 CH) stimulated the phagocytose of human primary granulocytes [29]. The results in Figure 1J are presented as percentages of change from Veh.-treated cells (set at 100%). The experimental conditions assessed here did not allow any effect of the MIM-3 on the granulocyte phagocytosis capabilities to be shown, when compared to the Veh.-treated cells.

### 2.2. MIM-4 Displays Slight Anti-Proliferative Effects on Colon Carcinoma Cells Cultivated in 2-Dimensions, under Starvation

**Figure 2:**
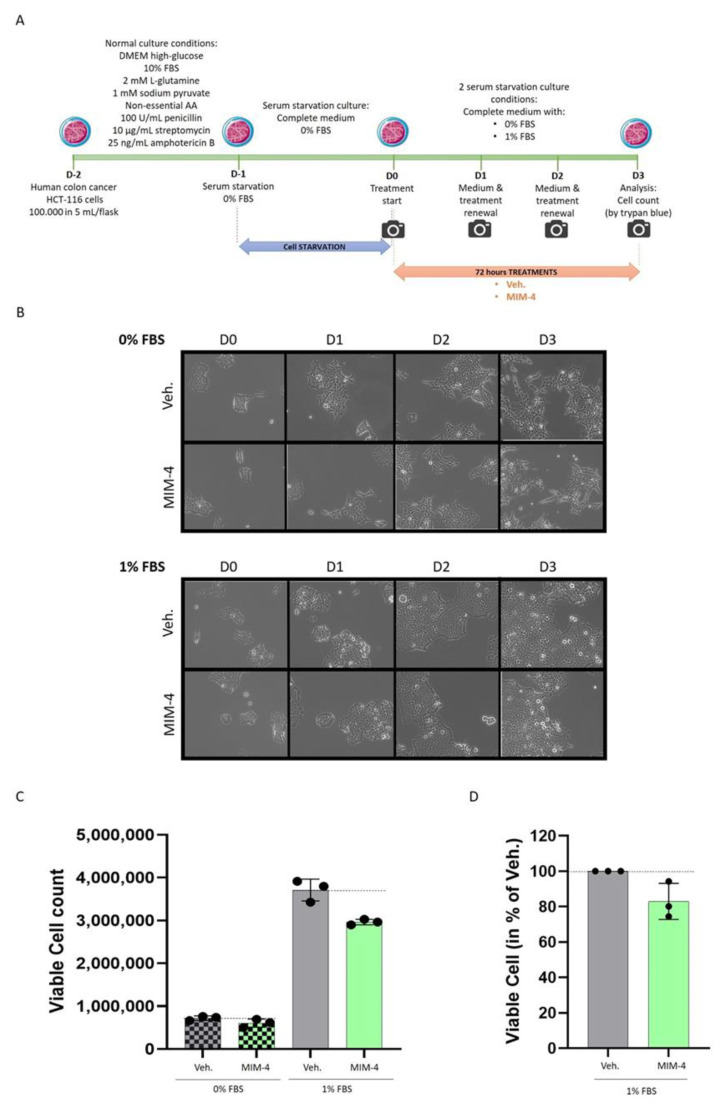
MIM-4 displays slight anti-proliferative effects on colon carcinoma cells cultivated in 2D, under starvation. (**A**) Representative scheme of the experimental protocol. HCT-116 cells were serum-starved for 24 h, and then treated with either MIM-4 or Veh. for a period of 72 h. The medium and the treatments were freshly renewed every day during the course of the treatment. The cells were kept in a starved state, by being supplemented or not with only 1% FBS. AA: amino acids. (**B**) Pictures were taken every day and representative pictures, taken with Evos XL Transmitted light microscope at the x 20 magnification, are presented. (**C**) Viable cells were manually counted by the trypan blue exclusion method at the end of the treatment. This experiment was performed once, and the results are presented as the mean ± SD of viable cell counts, repeated three times, as technical replicates. The black dot line was drawn in the graph to better visualize the effect of MIM-4 compared to its Veh. control. (**D**) The graph shows the results of the two experiments performed as the percentage of viable cells normalized on the respective Veh. (set to 100%). Each black dot represents the normalized results for one tested MIM-4 capsule.

The first experimental model chosen to assess the potential direct effect of MIM-4 on CRC cells was a conventional 2-dimensions (2D) model of CRC. Considering the presence of ULD of growth factors in MIM-4 formulation, hr-EGF (27 CH) and hr-TGF-β (27 CH) (see Table 1), and, as the rationale of this specific CH is the intention to disrupt EGF- and TGF-β-related-pathways in CRC cells, we wanted to avoid any exogenous supply of growth factors to the cancer cells, which are normally provided by fetal bovine serum (FBS). Briefly, HCT-116 cells were serum-starved for 24 h before treatment, and then treated with either MIM-4 or Veh. for a period of 72 h (Figure 2A). Pictures were taken every day to provide information on cell confluence prior to final quantification (Figure 2B). In the absence of serum, it was noticed that the cells displayed a more pronounced spindle-like shape that the MIM-4 treatment did not seem to affect. The viable cells were manually counted by the trypan blue exclusion method at the end of the treatment (Figure 2C). MIM-4 induced a decrease in cell viability under both serum-deprivation tested conditions. The effect is most noticeable in cells that have been cultured in 1% FBS. For this reason, the experiment was performed again in the 1% serum condition, and the results, combining the two experiments, normalized and expressed in % compared to the Veh., are shown in Figure 2D. MIM-4 was able to induce a decrease in HCT-116 cell viability of about 20% compared to Veh.-treated cells.

### 2.3. MIM-4 Displays Moderate Anti-Proliferative Effects on Colon Carcinoma Cells Cultivated in a 3-Dimensions-Spheroids’ Model

In order to test MIM-4 effects in a model that reproduces the interaction of cancer cells during the initiation of CRC tumor formation, and which, consequently, allows for a better reproduction of in vitro carcinogenesis than the usual 2D cell culture, a 3D spheroid model was established, using the same HCT-116 cells. In order to assess if the previous starved-conditions used in the 2D culture could also allow proper spheroid formation, a first 3D-type culture experiment was done, independently of any MIM-4/Veh.-treatment. For the proper setup of this spheroid model under serum deprivation, either the absence of serum concentration (0%) or several low serum concentrations (0.1%; 1%; and 2%) were tested. The experiment included the standard serum condition (10% FBS) as control, too.

**Figure 3:**
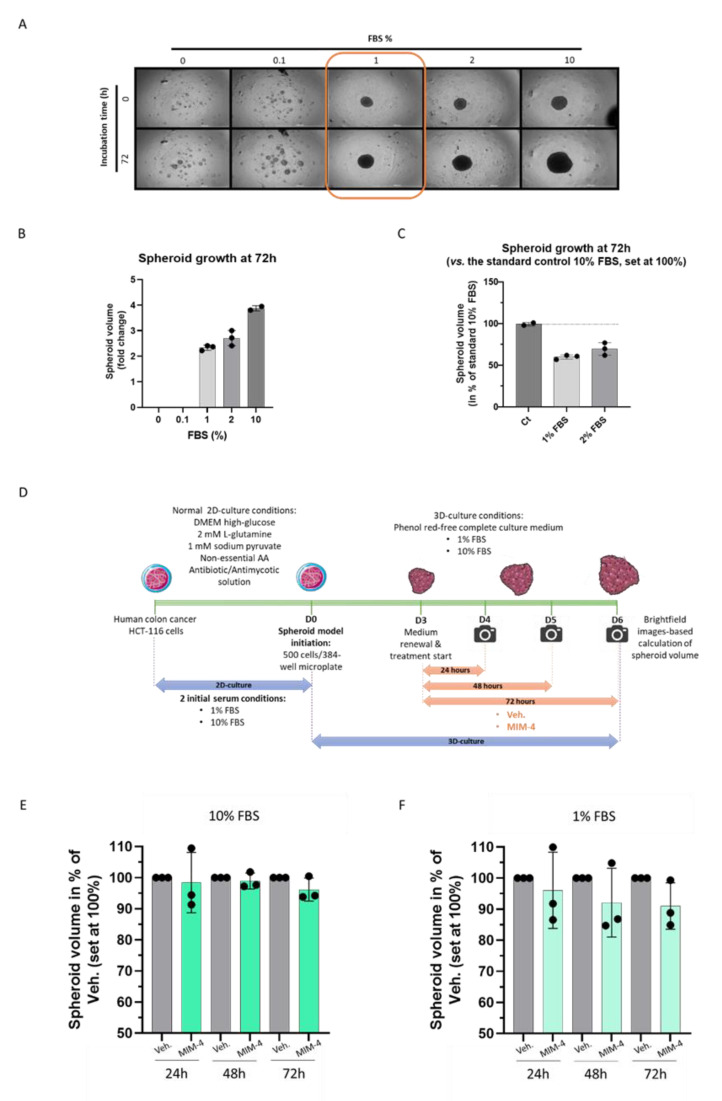
MIM-4 displays slight anti-proliferative effects on HCT-116 colon carcinoma cells cultivated in 3D-spheroids’ model. (**A**) Representative pictures of the spheroid growth regarding the different FBS percentages tested (ranging from 0 to 10% as shown in the legend) at the time of plating and after 72 h. The pictures were taken with an automated digital widefield microscope (BioTek Cytation 5). Three images in brightfield at distinct z-axis were taken with objective 4×. The orange box highlights the 1% serum concentration that is employed as a starved culture condition for the experiment illustrated and presented in (**D**,**E**). (**B**) HCT-116-derived spheroid growth after 72 h of incubation in the presence of various FBS% (ranging from 0 to 10% as shown in the graph legend). (**C**) The results are presented as the mean ± SD of spheroid volumes folds at 72 h normalized to the same spheroid volumes at D0, in each tested-condition. Each condition was performed in 3 technical replicates (black dots). (**D**) Scheme of the experimental protocol (**E**,**F**) Effect of MIM-4 on HCT-116-derived spheroid growth, either in 10% or in 1% FBS. The spheroid volume was calculated for each treatment-condition (MIM-4 or Veh.) and at either 24-, 48- or 72-h and expressed as a fold change of each endpoint-measure normalized to the initial spheroid volume at D0. Results are presented as the mean percentages ± SD of the Veh. -treated spheroids for each time-point. Each condition was performed in 3 technical replicates (black dots).

HCT-116 cells were seeded at 500 cells/wells in 384-well, clear round bottom ultra-low attachment microplates and incubated for 72 h in the above-mentioned serum concentrations. Pictures were taken at the end of the incubation period (Figure 3A) and the spheroid volume was calculated according to the method described in Section 4. As shown in Figure 3A,B, in the absence of serum, and at 0.1%, the cells were not able to aggregate into spheroids, whereas the tested concentrations ranging from 1% to 10% led to dose-dependent increase in spheroid growth, over the 72 h incubation time. Normalizing the data to the standard 10% FBS condition (set at 100%), the two more stringent starved conditions, which still permitted spheroids formation (1% FBS and 2% FBS), led to a volume reduction of about 40% and 30%, respectively (Figure 3C). As the 1% FBS was the minimum tested concentration leading to spheroid formation, this degree of starvation was further chosen for the following MIM-4 treatments (Figure 3A, orange boxed pictures).

As illustrated in Figure 3D, HCT-116-derived spheroids were thus formed either in 1% serum or in classical 10% over 72 h, then MIM-4 or Veh. were applied under those serum conditions for an additional 24, 48 or 72 h. The results showed that MIM-4 slightly decreased the spheroid volumes at 72 h, in both serum conditions, when compared with the Veh. control (Figure 3E,F). However, it can be noticed that MIM-4 exerted a clearer effect in reducing the spheroid volume in the 1% FBS culture conditions compared to the standard 10% FBS culture condition, that seemed to increase with the duration of treatment. As this experiment was performed once, inferential statistics were not performed. However, these results provide first-time evidence about the effect of MIM-4 on CRC cells, when these cells are self-organized into spheroids.

### 2.4. MIM-seq Displays Slight Anti-Tumor Effects in an In Vivo Subcutaneous Xenograft Model of Colon Carcinoma

With the potential use of MIM-seq as adjuvant therapy to ameliorate the response to standard oncological treatments and improve patients’ quality of life in mind, we wanted to assess its in vivo capabilities in reducing tumor progression, when employed alone, in a murine subcutaneous xenograft human CRC model. Moreover, the current study design aimed at reproducing, as closely as possible, the human situation in clinics, where, due to CRC’s silent nature [36], therapeutic management is frequently delayed and only starts when the tumor is already well established. This important parameter was taken into account in the experimental protocol and MIM-seq treatment was only initiated when mice-bearing tumor volumes were sufficiently developed.

**Figure 4:**
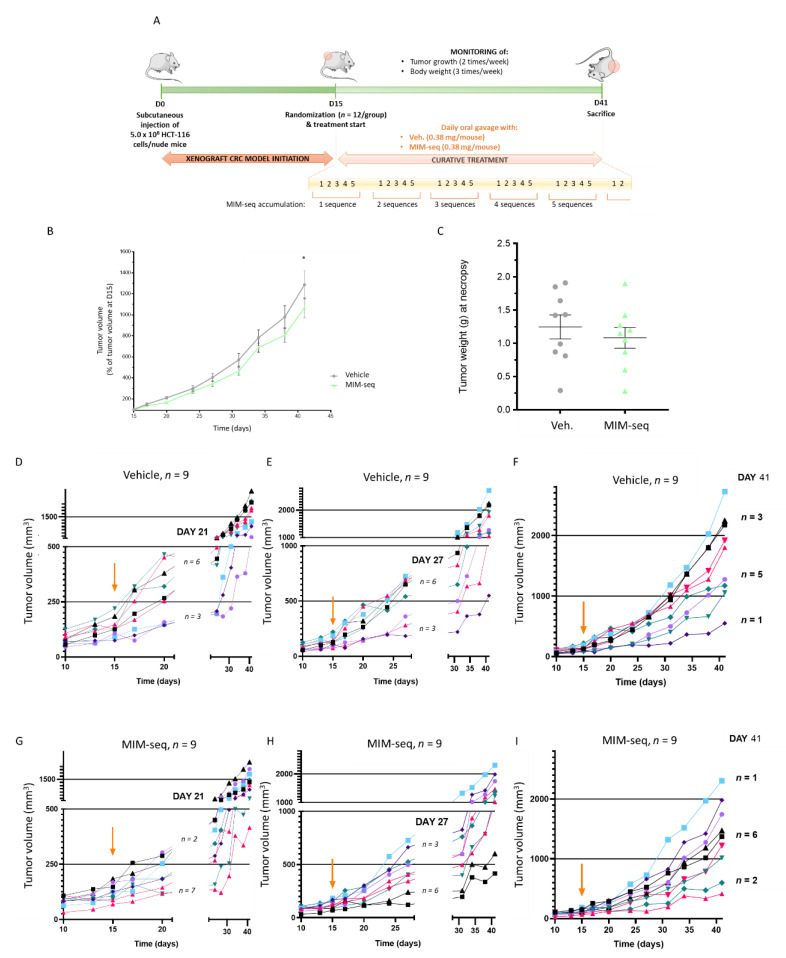
MIM-seq displays slight anti-tumor effects in a subcutaneous xenograft model of colon carcinoma. (**A**) Experimental scheme of the in vivo study. Briefly, HCT-116 cells were subcutaneously implanted into the left flank of each mouse on Day 0 (D0) and tumor-bearing animals were block-randomized into two groups (MIM-seq and Veh.) on Day 15 (D15). Starting from D15 to Day 41 (D41), each group was daily treated by oral gavage with 0.38 mg/100 μL water/mouse of Veh. or MIM-seq. Mice’s body weights and tumor growth were monitored during the treatment course. The experiment was ended on Day 41, when the animals were euthanized and the tumors were necropsied. (**B**) Tumor volume evolution is displayed from D15 to D41, as percentages of animal tumor volumes at D15 (*n* = 9 in each group) ± SD. The mean evolution of tumor growth between D15 and D41 was compared between groups with linear mixed models for repeated measures (*p* = 0.02 at D41). (**C**) Tumor weights were measured at necropsy (D41). The tumor weights (*p* = 0.48) are represented with scatter plots and mean ± SD. The two groups are compared with Wilcoxon-Mann-Whitney tests. (**D**–**I**) The graphs represent the evolution of tumor volume, from D10 to D41, in each mouse of Veh.-treated group (**D**–**F**) and MIM-seq-treated group (**G**–**I**). The orange arrow in graphs indicates the day of starting treatment (D15). The graphs were made to evaluate the evolution at different time windows: on D21 the cut-off was set at 250 mm^3^ (**D**,**G**), on D27 the cut-off was set at 500 mm^3^ (**E**,**H**), and on D41 two cut-offs were set at 1000 and at 2000 mm^3^ (**F**,**I**).

Briefly, 5.0 × 10^6^ cells from the HCT-116 cell line were subcutaneously implanted into the left flank of each mouse on Day 0 (D0). Tumor-bearing animals were block-randomized into two groups (MIM-seq and Veh., 12 animals in each group) on Day 15 (D15), when tumor volumes reached about 140 mm^3^ in both experimental groups. Starting from D15 to Day 41 (D41), each group was treated daily, by oral gavage with 0.38 mg/100 μL water/mouse of Veh. or MIM-seq. Mice body weights were checked three times per week and the tumor growth was monitored twice weekly after the start of the therapy. The experiment was ended on Day 41, when the animals were euthanized and the tumors were necropsied. In situ histological analysis of serial frozen tumor tissue sections were performed in order to quantify: (i) necrotic areas within the tumors, (ii) density of individual cell death (hematoxylin-eosin [H&E] staining), (iii) apoptosis (terminal deoxynucleotidyl transferase dUTP nick end labeling [TUNEL]) and (iv) cleaved caspase 3 immunohistochemistry in non-necrotic tumor tissues. The Figure 4A depicts a complete overview of the entire protocol.

After the start of treatment, mean animal weights of all study groups were evaluated. As illustrated in Figure S2A–C, the mean weight continuously increased from the beginning of the treatment until the study ended, and no major weight difference was observed between Veh. and MIM-seq groups.

Regarding the tumor volume evolution analysis, since three out of twelve mice per group were sacrificed before the end of the study for ethical reasons (ulcer development), we chose to only present the results related to the mice surviving until the last day (*n* = 9, in both groups). To properly evaluate the in vivo effect of MIM-seq, tumor volume data were expressed in percentage of the tumor volumes at the day of starting treatment (D15), the latter being set as 100% (Figure 4B and Figure S2D). In vivo tumor volume was slightly lower (about 20% less) in MIM-seq-treated mice than in the vehicle group. The comparison between the two groups with Wilcoxon-Mann-Whitney tests did not point out any difference in tumor volume at D41. Interestingly, the inferential statistics performed with linear mixed models for repeated measures, suitable in the context of repeated measures data and longitudinal studies, was significantly lower at D41 in the MIM-seq group compared to Veh.-treated group (*p* = 0.02). After necropsy, on D41, tumor wet weights were determined and a non-significant trend towards a decrease (*p* = 0.48) (Figure 4C and Figure S2E) was observed in the MIM-seq-treated group compared to the Veh.-treated one. The tumor volume after necropsy was also represented in Supplementary Figure S2F, in percentage of the Veh.-treated group. In addition, histopathology descriptive evaluation and in situ quantitative analyses were performed on tumor sections but no statistical difference was found between groups (Figure S3), amongst all the evaluated parameters (H&E, c-Caps-3, TUNEL, Ki67, and CD31).

In order to better describe the individual evolution of tumor volumes over time, in regards of the number of complete MIM-seq accumulation (see the yellow timeline, Figure 4A), the tumor volumes, expressed in mm^3^, at three different time windows, were illustrated for each Veh.-treated mouse (Figure 4D–F) and each MIM-seq-treated mouse (Figure 4G–I). The orange arrow in graphs indicates the day of start of treatment (D15).

On Day 21 (D21), one entire MIM-seq sequence (i.e., one round of MIM-1, MIM-2, MIM-3, MIM-4, and MIM-5 capsules), or 5 Veh.-capsules, were administered in each respective group (see the yellow timeline, Figure 4A). It can be observed that tumor volume stayed lower or equal to 250 mm^3^ in *n* = 7 out of the 9 mice of the MIM-seq-treated group, while *n* = 6 mice out of the Veh. control group developed a tumor having a volume higher than 250 mm^3^ (Figure 4D,G).

On Day 27 (D27), the mice of the MIM-seq-treated group had received the entire MIM-seq sequence twice, while mice of the control group received the Veh.. Figure 4E,H shows a picture of what happened in this second time window: an arbitrary cut-off being set at 500 mm^3^, *n* = 6 mice out of the MIM-seq-treated group had a tumor volume lower or equal to 500 mm^3^ and *n* = 3 mice, a tumor volume higher than 500 mm^3^. Inversely, in the control group only *n* = 3 mice had a tumor volume lower or equal to the cut-off, and *n* = 6 mice developed bigger tumors (>500 mm^3^).

On D41, MIM-seq-treated mice had received the entire sequence five times: to evaluate differences in tumor growth between MIM-seq- and Veh.-groups, two additional cut-offs were set; at 1000 and at 2000 mm^3^. While *n* = 2 mice out of the MIM-seq-treated group developed a tumor having a volume lower than 1000 mm^3^, only *n* = 1 mouse was observed in the control group. Then, looking at larger size volumes (>2000 mm^3^), only *n* = 1 of the MIM-seq-treated group developed it, compared with *n* = 3 mice in the control group (Figure 4F,I). No statistics were made with those raw data.

### 2.5. MIM-4 Could Act as an Adjuvant When Associated with Etoposide or Resveratrol, in an In Vitro 3D-Spheroid Model of Colorectal Cancer

In this study, we wanted to assess the potential anti-proliferative effect and the eventual toxicity of MIM-4, when combined with two conventional cancer compounds, the chemotherapeutic agent ETP, or the natural plant-derived RSV, by using the same 3D model of CRC as presented in Section 2.3 (HCT-116 cells-based spheroids), under serum standard condition (10% FBS).

**Figure 5:**
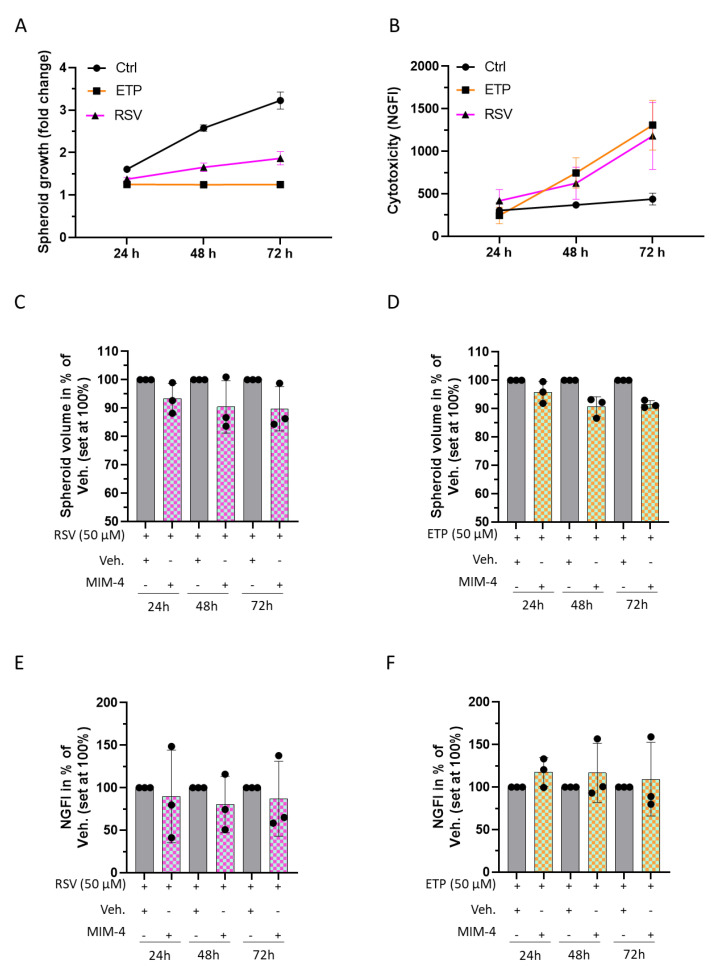
MIM-4 could act as an adjuvant when associated with etoposide or resveratrol, in an in vitro 3D-spheroid model of CRC. (**A**) Spheroids were cultivated in standard conditions and treated for 24, 48 or 72 h with either ETP (orange curve) or RSV (pink curve), at 50 µM each. Spheroids cultivated in standard conditions without any treatment were used as a positive control (Ctrl). Spheroid volumes were calculated and the growth evolution was represented as the fold change of the initial spheroid volume at the time of the treatment initiation. Results are presented as the growth mean ± SD for each condition (*n* = 3). (**B**) The cytotoxicity of both ETP and RSV was appraised in the same conditions as described in (**A**). The results are presented as the mean normalized green fluorescence intensity (NGFI) ± SD from CellTox green fluorescent dye, for each condition (*n* = 3). NGFI was calculated as described in Section 4.4.3. Spheroids were cultivated in the presence of RSV (**C**) or ETP (**D**) for 24, 48, or 72 h, concomitantly with either the Veh. (grey histograms) or MIM-4 (squared histograms) and the spheroid volumes were calculated in each condition. The results are presented as the mean ± SD of the volume percentage, the Veh. conditions being set at 100% (*n* = 3). The cytotoxicity of the association of RSV with either Veh. or MIM-4 (**E**) and the cytotoxicity of the association of ETP with either Veh. or MIM-4 (**F**) was appraised in the same assessed conditions as in (**B**,**C**). The results are presented as the mean ± SD of the NGFI percentage, the Veh.-conditions being set at 100% (*n* = 3).

First, CRC-spheroids were subjected to 50 µM of ETP or RSV for either 24, 48, or 72 h, when spheroid volumes were monitored. The cells were also incubated in the presence of the non-toxic CellTox green fluorescent dye, a DNA marker that only stains the DNA from dead cells, to quantify the cytotoxicity at the above-mentioned time-points. At the tested time-points and concentrations, ETP and RSV both affected the spheroid volumes, almost totally impeding their growth, and both increased the death of HCT-116 cells, compared to the control untreated spheroids (Figure 5A,B). As expected, the two agents are quite cytotoxic and exert strong anti-proliferative effects on CRC cells. Interestingly, when MIM-4 was combined with ETP, or with RSV, a slight reduction of the spheroid growth was observed compared to the vehicle control (Figure 5C,D). The cytotoxicity did not increase in MIM-4+ETP-treated cells compared to the vehicle control, rather, it seems that MIM-4 can slightly decrease the cell death of the cells treated with MIM-4 in association with RSV (Figure 5E,F). Even though the experiment was only performed once, the findings are promising and underline the need to further evaluate the potential benefit of MIM-seq as an adjuvant therapy.

## 3. Discussion

A large body of evidence demonstrates that the tumor microenvironment provides many signals that sustain colon tumorigenesis and CRC progression. Stromal cells and immune cells, particularly macrophages, produce huge amounts of cytokines, which exert proliferative and pro-survival effects on CRC cells [37]. It has been reported that macrophages represent up to 50% of the tumor mass. Macrophages originate from blood monocytes and are recruited to the tumor tissue. The presence of the so-called tumor-associated macrophages (TAMs) has been correlated with a bad prognosis [38]. However, TAMs can exert a dual supportive or inhibitory role on cancer, depending on the disease stage, or the tissue involved [38,39]. While some studies reported that high infiltration of TAMs correlates with better prognosis [40], their role in CRC is still controversial [39]. If more investigations are needed to understand their complex role in CRC, it is widely documented and accepted that inflammation is a hallmark of malignant CRC [18,41].

Considering the ability of macrophages to respond to different environmental cues, and bearing in mind their crucial role in the context of cancer and specifically CRC, we wanted to assess if the medicine could modulate the cytokine expression profile of macrophages under standard conditions and under different stimuli (20 ng/mL of IFN-γ, 5 ng/mL of IL-4, or 20 ng/mL IL-10), leading to distinct functional macrophages phenotypes, M1, M2a or M2c, respectively. Polarized macrophages differ in cytokine production and effector functions: the M1 phenotype is typically a producer of high levels of IL-12 and IL-23, while the M2 phenotype is characterized by low production of pro-inflammatory cytokines (see Figure S1). Due to its specific formulation encompassing IFN-γ (6 CH), together with other cytokines involved in immune responses (IL-2, IL-10, IL-12, and IFN-α) employed at LD or ULD, MIM-3 has been evaluated here.

As a whole, our results suggest that MIM-3, in allowing for an overall down-regulation of cytokine secretion levels in the differentially polarized macrophages (Figure 1B–I), could definitely act at a crossroads between immune and tumor cells. Indeed, according to our preliminary results, by managing a reduction in the amount of pro-inflammatory cytokines secreted by macrophages, MIM-3 could contribute to decreasing systemic inflammation in CRC. For instance, significant pairwise correlations have been found between some cytokines (TNF, IL-1β, IL-6, IL-8, IL-9, IL-10 or vascular endothelial growth factor [VEGF]) and between inflammatory markers, such as neutrophil-to-lymphocyte ratio, platelet-to-lymphocyte ratio, lymphocyte-to-monocyte ratio or fibrinogen in CRC patients, and those data, associated with tumor staging, could provide predictive information for CRC therapeutic management [41].

Amongst the MIM-3 composition, it is highly possible that IFN-α (3 CH) could have played a role in the anti-inflammatory responses of the treated macrophages. As a matter of fact, it has been shown that IFN-α-exposed monocytes produced significantly less IL-1β and TNF-α after toll-like receptor-8 (TLR-8) stimulation [42]. Interestingly, the same study has also reported that IFN-α-exposed monocytes reduced their IL12p70 production after having been stimulated with LPS plus IFN-γ as well, which resonates with the results that we presented here in Figure 1B. Finally, the presence of hr-IGF-1 at 27 CH within MIM-3 formulation could also have contributed to explain the effects that we observed on the secretion of TNF-α, IL-6 and IL-1β. Indeed, as this growth factor has been reported to stimulate the production of these cytokines in ex vivo monocytes, derived from human PBMCs [43], it is possible that its use at ULD may have exerted an inhibitory effect on IGF-1 function, in turn reducing the secretion of the three above-mentioned cytokines.

Phagocytosis has a pivotal role in the homeostatic clearance of dying cells and cell debris. In the context of cancer, phagocytosis could have both pro-tumorigenic or anti-tumorigenic consequences, depending on multiple factors [44] and, thus, may be important in regulating responses to anticancer therapies. Therefore, while keeping in mind the link between inflammation-related immune-responses and cancer, we wanted to investigate the effect of MIM-3 on the phagocytic capabilities of human granulocytes. In the present study, and under the tested experimental conditions, our results did not allow us to show any effect of the tested capsule on the phagocytic capabilities of human granulocytes (Figure 1J). Considering that previous evidence demonstrated enhanced phagocytosis in IFN-γ (4 CH)-treated granulocytes [29], and as MIM-3 contains IFN-γ (6 CH), our findings may appear unexpected. However, it is important to remember that MIM-3 is a complex MI medicine, which contains different active substances that can work in a synergistic or antagonistic manner towards a specific cellular response, depending on the context. In this regard, as IGF-1 was reported to be a potent stimulator of phagocytosis in human polymorphonuclear neutrophils [45], its use at 27 CH in MIM-3 could have perfectly antagonized the supposed IFN-γ (6 CH) effect. Moreover, two additional centesimal dilutions occur during the manufacturing protocol of IFN-γ (6 CH) compared to IFN-γ (4 CH), which could also provide further possible explanations to the observed difference in granulocyte responses. Taken together, our results perfectly illustrate that the assessment of any MIM’s biologic effects should be performed in a variety of immune cell subtypes, as well as in vivo, when possible, to fully appreciate the potential MIM’s effects in a context where the interactions between the diverse immune cells allows for integrative biological responses in the organism.

In addition to the evaluation of its immune-inflammation related effects, the anti-cancer potential of MIM-seq was also appraised in this study, in several models of CRC. Amongst the five capsules composing the sequential MIM-seq medicine, MIM-4 contains both EGF and TGF-β at 27 CH, as well as SNA targeting c-Fos- and c-Myc oncogenes (see the third column of Table 1, “composition of MIM-4”). For that reason, this capsule was chosen for all the in vitro study parts aimed at evaluating the direct effect of the medicine on CRC cell proliferation.

In order to find an in vitro experimental model that reproduced, as closely as possible, the posology of MIM-seq, a serum-starved model of CRC cells was established. Moreover, it had previously been reported that serum starvation sensitized CRC cells, including HCT-116 cells, resulting in an increased effect of anticancer agents [46]. The in vitro condition of serum starvation, by reducing the levels of growth factors, induces stress in both normal and cancer cells. Normal cells adapt and respond by downregulating the expression of proliferation-stimulating signaling pathways, allowing them to reduce their basal cellular metabolism and to enter into a reversible non-proliferating quiescent status [47]. Cancer cells respond by adapting and reprogramming their metabolism to maintain their continuous proliferation [48]. Interestingly in the present study, it was possible to better appreciate the anti-proliferative effect of MIM-4 in a classical 2D cell model of CRC, under serum starvation (Figure 2B,C). Moreover, amongst all the FBS percentages tested in our starvation tolerance evaluation, 1% appeared to be the threshold under which no 3D-spheroid aggregation was possible (Figure 3A,B). Even if the spheroid volume was reduced by about 40%, compared with the one obtained within standard 10% FBS culture (Figure 3C), this model still allowed us to highlight the anti-proliferative effects of MIM-4, even after only 24 h of incubation, compared with the Veh. condition (Figure 3F).

The results obtained in our in vitro models of HCT-116 cells could be directly related to the specific composition of MIM-seq. Indeed, with regards to CRC, several genes, such as *epidermal growth factor receptor* (*EGFR*), *c-Fos*, *c-Myc*, *RAS* family, *Notch-1*, *transforming growth factor receptor* (*TGFBR*) *1*, and *TGFBR2*, have been identified as crucial regulators involved in the proliferation and invasion processes of these specific cancer cells [49]. Keeping this in mind, and regarding the lack of growth factors in the media due to the starved context, the presence of EGF at the ULD of 27 CH could have reinforced the inhibition of the EGFR pathway, which is rightly involved in cancer-cells metabolism. Indeed, it has been reported that the EGFR downstream signalization cascade leads to the activation of various transcription factors involved in malignant transformation and tumor progression, typically through increased cell proliferation, prolonged survival, resistance to apoptosis, angiogenesis, invasion, and metastasis [50,51]. Moreover, as TGF-β is also an EGFR ligand, its presence at 27 CH in MIM-4 could also have acted synergistically with EGF (27 CH).

Another purpose of the current study was to evaluate the potential effects of the complete MIM-seq on tumor growth in an in vivo xenograft model of CRC. As this model is designed by subcutaneous injection of human cancer cells in immune deficient animals, it allowed us to properly evaluate the potential effects of drugs in neoplastic cells which maintain the same genetic and molecular profile to the patient-derived tumor cells [52]. Taking into account the silent nature of CRC, which can remain undetected for a long time in a large number of patients [36], the strategy employed in this study was to start MIM-seq treatment only at D15, when mice-bearing tumor volumes reached about 140 mm^3^ in both experimental groups. By doing so, the potential therapeutic effect of MIM-seq was thus appraised when administered in an already pre-established cancer, better mimicking the late stages at which CRC therapeutic management often starts.

The results were coherent with the first pieces of evidence obtained in vitro with MIM-4, as the integrality of the sequential MIM-seq, when administered to mice more than five times in a row after the initiation of the CRC, led to a significant (*p* = 0.02) reduction in tumor volume of about 20% compared to the controls (Figure 4B). However, as the intra-group variability for tumor volume in live animals was quite high, an in-depth analysis about the sequentiality effect of MIM-seq over the course of the mice treatment was also performed. Thus, in order to better describe the individual effects of one unique MIM-seq sequence on tumor volume progression, data were analyzed on D21, D27 and D41, as they corresponded to the administration of one complete MIM-seq sequence, once, twice, and 5 times, respectively (Figure 4D–I). At each time point, different cut-offs for tumor volume were set, and the results revealed a very interesting trend, as an inhibitory effect on tumor growth was already observed after the administration of the first five capsules of MIM-seq (Figure 4D,G). In line with these results, many more mice kept a tumor volume below 500 mm^3^, after having received two complete MIM-seq, than those which were administered with the Veh. (Figure 4E,H).

As encouraging as the MIM-seq effect obtained in our subcutaneous model is, we remain aware that several factors inherent to CRC development were not taken into account in this study. The burden of the metastatic spreading for instance, which is often located to the liver in CRC patients [53], or the cachexia associated with more advanced stages of the disease, were not assessed in the present work [54]. Moreover, intestinal immunity plays a paramount role in CRC prevention, as a statistically significant reverse correlation between the quantity of Peyer’s patches and the development of intestinal polyps has been shown in Apc^Min/+^ mice [55]. In this regards, orthotopic models of CRC, as they better reproduce the original tumor microenvironment than subcutaneous xenografts, would also be other promising tools to study the MIM-seq effects within the original tumor niche, as they would allow interactions between colon mucosa cells, cancer cells and immune cells as well [56].

It is also worth discussing the fact that our in vivo study was performed in the naval medical research institute (NMRI) nude mice strain, which is characterized by a severe, but not absolute, immune-deficiency, resulting in the lack of T cell responses, while still keeping intact the innate side of immunity (i.e., NK cells and macrophages) [57]. Interestingly enough, a study reported that innate immune reactions from nude mice were featured with an endogenous cytotoxic activity even higher than the one from euthymic animals [58]. In such a context, it is, thus, possible that the multi-targeted effects of MIM-seq, that we highlighted in the current in vitro part of the study could have operated in vivo, acting on both the remaining active cells from the innate side of the immunity and the cancer cells as well. Moreover, from a mechanistic standpoint, MIM-seq also employs mitogenic factors, such as epidermal growth factor (EGF), transforming growth factor-β (TGF-β) and fibroblast growth factor (FGF) 2 at 27 CH (ULD). Previous preclinical evidence has highlighted the inhibitory effects induced by ULD-based MI medicines [20,25,26,27,30]. Accordingly, it is possible that the active substances present in MIM-seq at the ULD of 6 CH and beyond, may reduce the expression of those mitogenic factors, which are crucial cancer-related signaling pathways, rightly overexpressed in several cancers, including CRC [50].

While tumor volume measurement by caliper is adequate for subcutaneous tumors, we are also perfectly aware that a lack of homogeneity was present in our model, and another more accurate way of monitoring tumor progression could have been employed. For instance, in live animals, and especially in orthotopic CRC models, in which tumor is not easily accessible, most advanced imagery techniques are used, such as magnetic resonance imaging, computed tomography, positron emission tomography, fluorescence imaging, bioluminescence imaging, or photoacoustic tomography [59]. As future perspectives, imaging techniques could also possibly be employed to follow the engraftment and the progression of 3D-CRC-derived spheroids in mice, as it has been done nicely by Tachibana et al., who also reported the uniformity of their radiation-crosslinked gelatin hydrogel-based transplantation scaffold model regarding tumor shape and timing of the formation of submillimeter CRC xenografts [60]. In any case, all the encouraging effects of either MIM-4 in vitro or MIM-seq in vivo as a single agent pave the way for assessing its effect, when associated with other therapeutic compounds.

Regarding the fact that MI medicines use, as a part of their formulations, LD and ULD of cytokines, the last objective of this study was to assess the potential effect of MIM-seq as a gentle adjuvant immune-therapy. The comprehensive composition of MIM-seq includes hr-IFN-α at 3 CH in association with hr-IL-2 at 6 CH. These two cytokines, when combined and associated with a conventional chemotherapy medicine, have already been reported to exert synergistic beneficial effects in patients suffering from metastatic renal carcinoma [61]. Moreover, MIM-seq also contains hr-IL-12 at 4 CH. Preclinical studies have demonstrated that IL-12 has remarkable antitumor effects against several cancers [62,63,64], and its ability to promote the intra-tumoral infiltration of natural killer (NK) cells has been suggested as one of the mechanisms underlying its antineoplastic effect over CRC [65]. It can thus be hypothesized that the presence of this cytokine at LD in MIM-seq might add a value in enhancing the synergy between the other active substances, and the potential benefits of cancer treatment. With the same rationale, the combination of several chemotherapeutic agents has already been done in the context of CRC. For instance, the association of Cisplatin (CIS) and ETP has been assessed in advanced CRC, and even if this combination seemed to be additive or synergistic, the objective remission rate was rather low, and hematologic toxicity was reported as one of its adverse effects [4], highlighting the need for other alternatives.

Etoposide derived from podophyllotoxin, which is a toxin found in the American Mayapple, was approved for cancer therapy in 1983 by the U.S. FDA [66]. By targeting the DNA topoisomerase II, ETP leads to the production of DNA breaks, causing DNA damage and consequent cell death [67]. Thus, in general, highly proliferative cells are the most sensitive cells affected by this agent. In the particular context of CRC cells, previous findings have demonstrated that ETP reduces the viability of HCT-116 cells [68]. Accordingly, we found that ETP (50 µM)-treated spheroids were strongly impacted by the treatment, as cytotoxicity was seen to be increased and spheroid growth impaired at 24, 48 and 72 h (Figure 5A,B). Coherently with prior studies reporting the lower toxicity of RSV, compared to genotoxic agents, such as ETP [69], and after testing the same concentration for both agents, we found that the effect on the two measured parameters, was stronger in ETP-treated spheroids (Figure 5A,B).

The results of the spheroids treated with MIM-4 in combination with ETP, reported in Figure 5D, revealed an early growth reduction, which, while slight, was already noticeable at 24 h, and more pronounced over time, especially at 48 and 72 h, compared to the corresponding control (Vehicle and ETP-treated spheroids). This evidence, while preliminary, opens new perspectives about the potential use of MIM-4 as an adjuvant medicine in patients suffering from CRC and treated with chemotherapy.

In parallel, due to their promising health benefits, natural polyphenols, such as RSV, have also been extensively researched as other alternatives to chemotherapy but again, their toxicity and side effects issues still need to be addressed [5]. Particularly in CRC, the anti-cancer properties of RSV are promising and are under investigation in clinical studies [70]. In addition to its anti-cancer effects, RSV also displays a wide range of pharmacological activities, including neuroprotection, antioxidation and anti-inflammatory effects [71]. The latter have been documented, notably, in RAW264.7 murine macrophages, in which it reduced the secretion of pro-inflammatory factors, such as IL-1β, IL12p70, TNF-α and IL-6 [72]. Moreover, and interestingly enough, its remarkable inhibitory effects towards the secretion of TNF-α and IL-6 have also been demonstrated in RSV-treated human monocyte-derived inflamed macrophages from both M1 and M2 phenotypes [73]. The fact that MIM-3 also displayed similar inhibitory effects on the secretion of these factors in the same human CD14^+^-derived polarized macrophages (Figure 1D,G) deserves to be highlighted. Regarding the intended use of MIM-seq as an adjuvant therapy for cancer treatment, such results open the door for its possible effect in sustaining RSV anti-inflammatory properties.

In the context of CRC, RSV exhibited the ability to interfere with the signaling pathways involved in the initiation and progression of this cancer, including silent mating type information regulator two homolog 1 (SIRT1), P53, P21, AMP-activated protein kinase (AMPK), reactive oxygen species (ROS), cyclooxygenase (COX) 2, nitric oxide (NO), caspases, wingless-related integration site (Wnt), TNFs, and nuclear factor kappa-light-chain-enhancer of activated B cell (NF-κB) signaling [70]. Interestingly, while COXs are important regulators of inflammation and angiogenesis, they are also implicated in carcinogenesis, as the overexpression of COX-2 has been reported as one of the causative factors for CRC development [74]. RSV and RSV-derived compounds, such as RSV-aspirin hybrid compounds, by targeting COX activity, are also considered potent intestinal anti-inflammatory and anti-tumor drugs [75,76].

However, the chemotherapeutic doses that efficiently induce oxidative stress, and apoptosis, and which reduce proliferative capacities in cancer cells, were shown to be cytotoxic to normal healthy cells [77]. About this, it has been observed that RSV decreased fibroblast viability in a dose-dependent manner [78], as well as induced cell senescence while inhibiting cell renewal in mesenchymal stem cells, at concentrations above 5 mM [79]. Indeed, toxicity and adverse effects were reported in patients assuming RSV [77].

Interestingly, in our model of spheroids treated with MIM-4 in association with RSV, an anti-proliferative effect was noticed, without increment in the number of cell deaths (Figure 5C–E). Thus, these data have revealed that MIM-seq could work in association with RSV in enhancing the anti-proliferative effect, whilst reducing cytotoxicity.

Having chosen to perform MIM-4 tests in both 2D and 3D in vitro models, this could be considered as one of the strengths of this study, as the kind of cell culture type influences drug responses. For instance, it has indeed been reported that the 3D-cultivation of CRC cell lines (including HCT-116) increased their chemoresistance to the anti-metabolite 5-Fluorouracil and the alkylating agent Cisplatin, compared with 2D cultures [80]. These data are quite interesting as they highlight the fact that the active ingredients of MIM-4 are still able to reach the cancer cells when assembled into spheroid-structures, resulting in the potentiation of the RSV and ETP effects (Figure 5).

The overall results obtained in the 3D spheroid model of CRC, while still needing additional investigations, are encouraging and merit catching the interest of the scientific community about the potentials of MI as adjuvant therapy in the context of CRC.

## 4. Materials and Methods

### 4.1. Tested Item and Experimental Control

The tested MI medicine, MIM-seq, is manufactured by Labo’Life España, as previously described [28,29], and was provided for investigational purpose.

MIM-seq is presented as a sequence of 5 capsules, each one having a specific composition, intended to be taken daily, following the numerical order indicated on the blister. Due to confidentiality issues arising from intellectual property, the composition of each specific capsule has not been disclosed.

The composition of MIM-seq has been chosen, taking into account the characteristics of this specific study’s endpoints and, consequently, is characterized by cytokines, growth factors and SNA^®^ (expressed as CH), and by the use of total DNA and RNA (extracted from *Pinus halepensis* and *Foeniculum vulgare*, respectively), expressed as Korsakovian dilution (K). The overall composition of MIM-seq, also described in Table 1, is as follows: hr-IL-1β (9 CH), hr-IL-2 (3 CH), hr-IL-6 (9 CH), hr-IL-7 (4 CH), hr-IL-10 (27 CH), hr-IL-12 (4 CH), hr-epidermal growth factor (EGF) (27 CH), hr-IGF-1 (27 CH), hr-basic fibroblasts growth factor (FGF2) (27 CH), hr-IFN-α (3 CH), hr-IFN-γ (6 CH), hr-TGF-β (27 CH), hr-TNF-α (5 CH), RNA and DNA (6, 12, 30, 200 K), SNA^®^-HLA I, SNA^®^-HLA II (18 CH), SNA^®^-C1a01, SNA^®^-C1b01, and SNA^®^-C1c01 (18 CH). SNA^®^-HLA I and SNA^®^-HLA II were designed to target human leukocyte antigen (HLA)-I and HLA-II. SNA^®^-C1a01, SNA^®^-C1b01, and SNA^®^-C1c01 are intended to specifically target the oncogenes c-Myc and c-Fos.

The following Table 1 describes the whole composition of MIM-seq and the composition of the two in vitro tested capsules (the third and the fourth capsules of MIM-seq, MIM-3 and MIM-4, respectively).

The Veh. pillules used in the study as controls are manufactured by Labo’Life Belgium. Previous publications describe how those vehicle controls are produced, in order to provide a suitable control for preclinical research (for both in vitro and in vivo evaluations) [20,26,27,28,29]. For all the in vitro experiments, MIM-3, MIM-4, or the Veh. pillules were freshly diluted in culture medium to reach the final sucrose-lactose concentration of 11 mM (corresponding to the content of one capsule in 100 mL). Earlier evidence demonstrated that this concentration is suitable to evaluate the in vitro biological effect of MI medicines [22,23,28,29].

### 4.2. Phagocytosis

Granulocytes were isolated from total peripheral blood of one healthy donor, after Ficoll^®^ gradient separation. The cells were grown in RPMI-1640 medium supplemented with L-glutamine 2 mM, Penicillin 100 U/mL-Streptomycin 100 μg/mL and Bovine Serum Albumin (BSA) 0.1% at 37 °C and 5% CO_2_. The experiment was performed in 96-well plates; MIM or the positive control fMLP 10 µM (used as a phagocytosis inducer) were preincubated with the cells for 10 min at 37 °C. In order to include the proper experimental control, the Veh. was run in parallel too. Fluorescent beads (Molecular Probes™ FluoSpheres™ Carboxylate-Modified Microspheres, 1.0 μm, yellow-green fluorescent 505/515) were then added and incubated with the cells for 45 min. Untreated non-beads-incubated cells were used as a negative control. All the conditions were performed in triplicate. After the incubation period, cells were rinsed in Phosphate Buffered Saline (PBS)/(BSA) 0.1% and centrifugated. Acquisitions were realized with 10,000 cells/replicate on a BD FACSVerse^TM^ cytometer. Regarding the fact that fluorescence-increase in the FITC channel is proportional to the phagocytosed beads (emission beads wave length = 515 nm), the results were first expressed as FITC-positive percentages, then expressed in percentage of the Veh.-treated cells, set as 100%.

### 4.3. Macrophage Cytokine Secretion Evaluation

CD14^+^ cells were freshly isolated from PBMCs obtained from the blood of one healthy donor thanks to the Miltenyi kit (# ref: 130-050-201, Miltenyi Biotec, Bergisch Gladbach, Germany). Briefly, the cells were seeded at the density of 50,000 cells/well in 96-well plates on Day 0 and were cultivated in RPMI 1640 supplemented with 2% inactivated human serum, 1 mM non-essential amino acids, 1 mM pyruvate, 2 mM L-glutamine, 10 mM HEPES buffer and 50 ng/mL M-CSF. Cells were treated with vehicle or MIM-3 from Day 1 until Day 7, both medium and treatments being renewed on Day 3 and Day 5. The experimental scheme is shown in Figure 1B. For cytokine secretion assessment, the cells were stimulated with LPS on Day 6 (100 ng/mL) and harvested on Day 7. Supernatants were collected after cells centrifugation, and cytokine measurement was directly performed by ELISA (LEGENDplex^TM^, BioLegend^®^, San Diego, CA, USA) on fresh supernatants. The assessed secreted-cytokine’s panel included IL-23, TNF-α, IL12p40, IL-6, arginase, TARC and IL-1β. Because only one experiment was conducted, no statistical inference has been performed. The results of the cytokine secretion are presented as percentages of the mean ± SD of *n* = 3 replicates per condition; the cytokine secretion in the Veh.-treated cells being set at 100%, for each differentiation condition.

### 4.4. Colon Cancer In Vitro Models

#### 4.4.1. Experiments Performed on Classical Monolayer Cultured HCT-116 Cells

Two days prior to treatment, HCT-116 colon cancer cells were suspended in complete medium DMEM high-glucose (ref: D5671, Sigma-Aldrich, Saint-Louis, MI, USA), supplemented with 10% FBS (Euroclone, Pero, Italy; #ECS0180L), 2 mM L-glutamine (ref: X0550, VWR International, Radnor, PA, USA), antibiotic antimycotic solution (AA, ref: L0010, VWR International), 1 mM sodium pyruvate (ref: S8636, Sigma-Aldrich) and non-essential amino acids (ref: M7145, Sigma-Aldrich), and counted using Cell Countess II (Life Technologies, Carlsbad, CA, USA). 100,000 cells in 5 mL were plated in three T25 flasks with vented filter. After 24 h, medium was removed and substituted with 5 mL no-serum medium (complete medium without any FBS addition). After 24 h of serum starvation, medium was replaced with fresh medium (supplemented or not with 1% FBS) and the cells were treated with either the Veh. or MIM-4. After 1 day and 2 days from start of treatment, medium and treatments were renewed and phase contrast images at 20× objectives were taken using EVOS XL Core Imaging System (Thermo Fisher Scientific, Waltham, MA, USA). After 3 days from start of treatment, pictures were taken again and cells were detached by trypsinization and counted with trypan blue. Briefly, after trypsinization, cells were suspended in 1.3 mL of medium. Then, 16 μL of suspension was mixed with 16 μL trypan blue solution 2×. After 5 min, 10 μL of solution were loaded in each chamber of a NanoEnTek cell counting slide. Cell suspensions were counted using Cell Countess II (Life Technologies).

#### 4.4.2. Initiation of the Spheroid Model

HCT-116 colon cancer cells were cultured in DMEM (ref: D5671, Sigma-Aldrich, Saint-Louis, MI, USA) supplemented with 10% FBS (Euroclone, Pero, Italy; #ECS0180L), 2 mM L-glutamine (ref: X0550, VWR International, Radnor, PA, USA), antibiotic antimycotic solution (ref: L0010, VWR International), sodium pyruvate (ref: S8636, Sigma-Aldrich) and non-essential amino acids (ref: M7145, Sigma-Aldrich). Cells were cultured at 37 °C, 5% CO_2_ humidified air. For the 3D tumor spheroid formation assay, cells were detached by trypsinization and suspended in fresh phenol red-free complete culture medium (Euroclone, LOBE12917F), with FBS percentages ranging from 0 to 10%, depending on the experiments. Then, 20 μL of cell suspension, containing 500 cells, were plated in each well of a 384-well black, clear round bottom ultra-low attachment spheroid microplate (Corning 3830, Corning, NY, USA). Plating of cells was performed with an automated liquid handling platform (Gilson Pipetmax, Gilson Incorporated, Middleton, WI, USA). Plates were incubated at 37 °C and 5% CO_2_ humidified air to allow spheroid formation. After 72 h of incubation, spheroids were used for growth assay with the tested items, alone or in combination (Veh., MIM-4, RSV or ETP). During the treatment course, the final FBS percentages were either kept at 1% or 10% for the experiments involving Veh. and MIM-4 alone, while the ones including RSV and ETP treatments were only performed with 10% FBS.

#### 4.4.3. Kinetics of 3D Spheroid Growth and Cytotoxicity

Spheroids were treated with 10 μL of fresh medium containing MIM-4 or Veh. at proper dilution, supplemented with CellTox green fluorescent dye (Promega, Madison, WI, USA). CellTox green dye is a non-toxic DNA stain that is excluded from viable cells but stains the dead cells’ DNA. When the dye binds DNA in compromised cells, the dye’s fluorescent properties are enhanced; therefore, the fluorescent signal produced by the dye is proportional to the number of dead cells. Serial dilutions were prepared at a concentration 3x in 10 μL to obtain desired treatment concentrations in 30 μL of total medium. Three technical replicates were performed for all Veh. or MIM-4 treatments, whereas six technical replicates were performed for negative control (spheroids treated with medium) and positive control (spheroids treated with ETP 50 μM, or RSV 50 μM). Preparation of serial dilutions and treatment of spheroids were performed with an automated liquid handling platform (Gilson Pipetmax, Gilson Incorporated). Immediately after the start of treatment, plates were incubated at 37 °C and 5% CO_2_ in an automated digital widefield microscope with temperature and CO_2_ control system (BioTek Cytation 5, BioTek Instruments, Winooski, VT, USA). Three images in brightfield and green fluorescent protein (GFP) fluorescence at distinct z-axis were taken with objective 4x immediately after the start of treatment and at 24, 48 and 72 h of treatment.

#### 4.4.4. Data Analysis

Brightfield and GFP images at distinct z-axis (50 μm between each image) were processed and merged to obtain 3D images covering 150 μm of z-axis. Z-projected brightfield images were used to define spheroid area (further used for the determination of the spheroid volumes). Green fluorescence integral (GFI) in the delimited spheroid area, emitted by CellTox green dye (Promega) and representative of cell death, was quantified by Z-projected GFP images (further used for the cytotoxicity representations, expressed as normalized green fluorescence intensity [NGFI]).

Spheroid volume at each time point (V) was calculated as:

$$V=A\times \frac{4}{3}\sqrt{\frac{A}{\pi }}$$

where A is the measured area of the spheroid at the same time point.

Normalized Green Fluorescence Intensity (NGFI) of each spheroid, at each time point, was calculated as:

$$\mathrm{NGFI}=\frac{\mathrm{GFI}-{\mathrm{GFI}}_{0}}{A}$$

where GFI is green fluorescence integral at the analyzed time point, GFI_0_ is green fluorescence integral of the same spheroid at time point zero (immediately after start of treatment), and A is the area of the same spheroid at the analyzed time point [81,82].

### 4.5. Colon Cancer Animal Model

#### 4.5.1. Animal Housing and General Experimental Conditions

This animal study was approved by the Ethics Committee for Animal Experimentation and registered by the regional board Freiburg. Mice were handled according to the German animal welfare law and the GV-SOLAS guidelines. Health monitoring of the animal facility was done according to FELASA guidelines quarterly by examination of sentinel animals.

Female NMRI nude mice (Charles River Laboratories, Wilmington, MA, USA) 4–5 weeks of age were used in the in vivo experiments. Four animals per cage were housed in individual ventilated cages (Zoonlab GmbH, Castrop-Rauxel, Germany; air conditioned with 10–15 air changes per hour) under temperature (22 ± 2 °C), humidity (45–65%), and light (12-h light/dark cycle) controlled environment. At randomization, mice assigned to the same group (12 mice per group) were housed together. Animal behavior was monitored daily throughout the study. Deviation of the health status of the animals were documented and animals were individually euthanized by cervical dislocation, before study termination when ethical abortion criteria (e.g., tumor diameter ≥ 2 cm, tumor ulceration, body weight loss ≥20%, or signs of sickness) were reached.

#### 4.5.2. Cell Culture for the Generation of the In Vivo Model

HCT-116 tumor cells were derived from human CRC. Cells were grown in DMEM Glutamax 1 (Gibco by Life Technologies, Carlsbad, CA, USA) supplemented with 10% Fetal Calf Serum (FCS), (Bio&SELL GmbH, Feucht bei Nuremberg, Germany), 100 units penicillin/mL and 100 µg of streptomycin/mL (Capricorn Scientific GmbH, Ebsdorfergrund, Germany) at 37 °C in a 10% CO_2_ atmosphere. 70–90% confluent cultures were split passaged routinely using trypsin/EDTA (Capricorn Scientific GmbH).

#### 4.5.3. In Vivo Study Design

On Day 0, tumor cells (5.0 × 10^6^ cells in 100 µL PBS [Capricorn Scientific GmbH]) were subcutaneously implanted into the left flank of each mouse. Animal tracking and clinical signs were monitored with a daily inspection and documentation of anomalies. Body weights were checked three times weekly and tumor growth monitoring was performed two times weekly after start of therapy. Primary tumor volumes were determined by caliper measurement and tumor sizes were calculated according to the formula W2 × L/2 (L = length and W = the perpendicular width of the tumor, L > W).

On Day 15, tumor-bearing animals were block-randomized into 2 groups of 12 animals, each with a mean tumor volume of approximately 140 mm^3^. For block-randomization, a robust automated random number generation within individual blocks was used. Every day, from Day 15 to Day 41, sucrose-lactose pillules contained in one capsule of vehicle or MIM-seq (about 380 mg) were dissolved in 100 mL of water (corresponding to the sucrose-lactose final concentration of 11 mM) and 100 µL/mouse (corresponding to 0.38 mg/mouse) of this solution was administered by oral gavage.

Animals were euthanized by cervical dislocation and a necropsy was performed on Day 41. During necropsy, animals were weighed, primary tumors were collected, and wet weights determined. Primary tumor tissues were snap-frozen in liquid nitrogen, transferred to polypropylene and stored at −80 °C.

### 4.6. Statistical Analysis

The graphs in figures were performed with GraphPad Prism, Version 9.3.1 for Windows (GraphPad Software Inc., San Diego, CA, USA, accessed on 3 January 2022). Authors followed the recent recommendations that encourage performing descriptive statistics instead to make statistical inferences when the number of independent values is small. Indeed, no statistical inference has been performed to analyze the results of the in vitro studies presented here. In order to show the results in a transparent manner, when data were obtained from only one experiment replicates were plotted together with the mean ± SD in the graphs (Figure 1, Figure 2, Figure 3 and Figure 5). For the in vivo study, statistical analyses were performed using SAS software 9.4 (SAS Institute, Cary, NC, USA). The mean evolution of tumor growth between Day 15 and Day 41 (Figure 4B) were represented and compared between groups with linear mixed models for repeated measures (Day, group and interaction between day and group were the fixed effects and mouse was the random effect). To evaluate the difference between the two groups (Veh. and MIM-seq) in tumor weight measured at necropsy (at Day 41), (see Figure 4C) the Wilcoxon-Mann-Whitney test was performed with GraphPad Prism, Version 9.3.1. The levels of significance were set at *p* < 0.05.

## 5. Conclusions

Overall, the present study highlighted the dual effects of MIM-seq as a potential immunomodulatory agent and a potential antineoplastic agent, alone or in association with the chemotherapeutic drug ETP and the natural compound RSV. Indeed, as reported here, the MIM-3 capabilities, oriented towards a resolution of the LPS-induced pro-inflammatory cytokine secretion, in a model of CD14^+^-derived M0, M1 and M2 macrophages, could be transposed into a more global effect against the systemic inflammation that is generally associated with CRC. These results about potential MIM-seq roles from an immune perspective, were also reinforced by the fact that MIM-4 displayed a slight in vitro anti-cancer effect towards HCT-116 CRC cells. The entire MIM-seq, when administered in vivo in a murine subcutaneous xenograft model of CRC, was reported to slightly impair tumor growth, as tumor volumes were found to be significantly lowered by about 20% in the MIM-seq group compared with the Veh. one. Finally, used alone, in serum-deprived conditions, or in association with RSV or ETP, in standard serum cultured-conditions, MIM-4 was shown to moderately reduce the spheroid volume of 3D models of HCT-116 by about 10% compared to the vehicle control after 72 h. These results, while still preliminary, are promising and show that MIM-seq might exert anti-tumor effects and deserves further investigation to assess its potential as adjuvant therapy in the management of CRC.

## Figures and Tables

**Table 1:** Composition of the complete sequence of the MIM-seq (in vivo experiment) and the two tested capsules, MIM-3 and MIM-4 (in vitro experiments). CH: centesimal Hahnemannian; K: Korsakovian dilution; SNA^®^: specific nucleic acids.

MIM-seq Composition	MIM-3 Composition	MIM-4 Composition
**hr-IL-1β (9 CH)**	-	-
**hr-IL-2 (3 CH)**	hr-IL-2 (3 CH)	-
**hr-IL-6 (9 CH)**	-	-
**hr-IL-7 (4 CH)**	-	-
**hr-IL-10 (27 CH)**	hr-IL-10 (27 CH)	-
**hr-IL-12 (4 CH)**	hr-IL-12 (4 CH)	hr-IL-12 (4 CH)
**hr-IFN-α (3 CH)**	hr-IFN-α (3 CH)	-
**hr-IFN-γ (6 CH)**	hr-IFN-γ (6 CH)	-
**hr-TNF-α (5 CH)**	-	hr-TNF-α (5 CH)
**h-EGF (27 CH)**	-	h-EGF (27 CH)
**h-bFGF (27 CH)**	-	-
**hr-IGF-1 (27 CH)**	hr-IGF-1 (27 CH)	-
**hr-TGF-β (27 CH)**	-	hr-TGF-β (27 CH)
**DNA (6-12-30-200 K)**	-	DNA (6–12 K)
**RNA (6-12-30-200 K)**	RNA (30–200 K)	-
**SNA^®^-C1a01 (18 CH)**	-	SNA^®^-C1a01 (18 CH)
**SNA^®^-C1b01 (18 CH)**	-	SNA^®^-C1b01 (18 CH)
**SNA^®^-C1c01 (18 CH)**	-	SNA^®^-C1c01 (18 CH)
**SNA^®^-HLA I (18 CH)**	SNA^®^-HLA I (18 CH)	-
**SNA^®^-HLA II (18 CH)**	-	-

## Supplementary Materials

The following are available online at https://www.mdpi.com/article/10.3390/ijms23116059/s1.

## Abbreviations

2D:	2-dimensions
3D:	3-dimensions;
AA:	amino acids;
AMPK:	AMP-activated protein kinase;
BSA:	bovine serum albumin;
CH:	centesimal Hahnemannian
COX:	cyclooxygenase
CRC:	colorectal cancer;
CTLs:	cytotoxic T lymphocytes;
DNA:	deoxyribonucleic acid
EGF:	epidermal growth factor;
EGFR:	epidermal growth factor receptor
ELISA:	enzyme linked immunosorbent assay;
EMA:	European Medicines Agency;
ETP:	etoposide;
FBS:	fetal bovine serum;
FCS:	fetal calf serum;
FDA:	Food and Drug Administration;
FGF2:	fibroblast growth factor 2;
fMLP:	N-formyl methionyl-leucyl-phenylalanine;
GFI:	green fluorescence integral;
GFP:	green fluorescent protein;
GM-CSF:	granulocyte-macrophage colony-stimulating factor;
H&E:	hematoxylin and eosin;
HLA:	human leukocyte antigen;
hr:	human recombinant;
IFN:	interferon;
IHC:	immunohistochemistry;
ILs:	interleukins;
K:	Korsakovian dilution;
LD:	low dose;
LPS:	lipopolysaccharide;
M1:	M1 macrophage;
MCH:	major histocompatibility complex;
M-CSF:	macrophage colony-stimulating factor;
MI:	micro-immunotherapy;
MIM:	micro-immunotherapy medicine;
NF-κB:	nuclear factor kappa-light-chain-enhancer of activated B cells;
NGFI:	normalized green fluorescence intensity;
NK:	natural killer;
NMRI:	naval medical research institute;
NO:	nitric oxide;
PBMCs:	peripheral blood mononuclear cells; PBS: Phosphate buffer saline;
PFA:	Paraformaldehyde;
P/S:	penicillin/streptomycin;
RNA:	ribonucleic acid;
ROS:	reactive oxygen species;
RSV:	resveratrol;
RT:	room temperature;
SIRT1:	silent mating type information regulator two homolog 1;
SKP:	serial kinetic process;
SNA^®^:	specific nucleic acids;
TAMs:	tumor-associated macrophages; TARC: thymus and activation-regulated chemokine;
TGF-β:	transforming growth factor-β;
TGFBR:	transforming growth factor receptor;
TLR:	toll-like receptor;
TNF-α:	tumor necrosis factor-α;
TUNEL:	terminal deoxynucleotidyl transferase dUTP nick end labeling;
ULD:	ultra-low dose;
VEGF:	vascular endothelial growth factor;
Veh:	vehicle;
Wnt:	wingless-related integration site;

## References

[1] Recio-Boiles A., Cagir B. (2022). Colon Cancer.

[2] Schmoll H.J., Van Cutsem E., Stein A., Valentini V., Glimelius B., Haustermans K., Nordlinger B., van de Velde C.J., Balmana J., Regula J. (2012). ESMO Consensus Guidelines for management of patients with colon and rectal cancer. A personalized approach to clinical decision making. Ann. Oncol..

[3] Van Cutsem E., Cervantes A., Adam R., Sobrero A., Van Krieken J.H., Aderka D., Aranda Aguilar E., Bardelli A., Benson A., Bodoky G. (2016). ESMO consensus guidelines for the management of patients with metastatic colorectal cancer. Ann. Oncol..

[4] Golshani G., Zhang Y. (2020). Advances in immunotherapy for colorectal cancer: A review. Ther. Adv. Gastroenterol..

[5] Johdi N.A., Sukor N.F. (2020). Colorectal Cancer Immunotherapy: Options and Strategies. Front Immunol..

[6] Shankaran V., Ikeda H., Bruce A.T., White J.M., Swanson P.E., Old L.J., Schreiber R.D. (2001). IFNγ and lymphocytes prevent primary tumour development and shape tumour immunogenicity. Nature.

[7] Wrangle J.M., Patterson A., Johnson C.B., Neitzke D.J., Mehrotra S., Denlinger C.E., Paulos C.M., Li Z., Cole D.J., Rubinstein M.P. (2018). IL-2 and Beyond in Cancer Immunotherapy. J. Interferon. Cytokine Res..

[8] Khan W.A. (2019). Autoantibodies and Cytokines.

[9] García-Martínez E., Smith M., Buqué A., Aranda F., de la Peña F.A., Ivars A., Cánovas M.S., Conesa M.A.V., Fucikova J., Spisek R. (2018). Trial Watch: Immunostimulation with recombinant cytokines for cancer therapy. Oncoimmunology.

[10] Vacchelli E., Aranda F., Bloy N., Buqué A., Cremer I., Eggermont A., Fridman W.H., Fucikova J., Galon J., Spisek R. (2016). Trial Watch—Immunostimulation with cytokines in cancer therapy. OncoImmunology.

[11] Li J., Huang L., Zhao H., Yan Y., Lu J. (2020). The Role of Interleukins in Colorectal Cancer. Int. J. Biol. Sci..

[12] Marabondo S., Kaufman H.L. (2017). High-dose interleukin-2 (IL-2) for the treatment of melanoma: Safety considerations and future directions. Expert Opin. Drug. Saf..

[13] Wolkenstein P., Chosidow O., Wechsler J., Guillaume J.C., Lescs M.C., Brandely M., Avril M.F., Revuz J. (1993). Cutaneous side effects associated with interleukin 2 administration for metastatic melanoma. J. Am. Acad. Dermatol..

[14] Kirkwood J.M., Resnick G.D., Cole B.F. (1997). Efficacy, safety, and risk-benefit analysis of adjuvant interferon alfa-2b in melanoma. Semin. Oncol..

[15] Atkins M.B., Lotze M.T., Dutcher J.P., Fisher R.I., Weiss G., Margolin K., Abrams J., Sznol M., Parkinson D., Hawkins M. (1999). High-Dose Recombinant Interleukin 2 Therapy for Patients with Metastatic Melanoma: Analysis of 270 Patients Treated Between 1985 and 1993. J. Clin. Oncol..

[16] Coussens L.M., Werb Z. (2002). Inflammation and cancer. Nature.

[17] Papiernik M., de Moraes M.L., Pontoux C., Vasseur F., Pénit C. (1998). Regulatory CD4 T cells: Expression of IL-2R alpha chain, resistance to clonal deletion and IL-2 dependency. Int. Immunol..

[18] Tuomisto A.E., Mäkinen M.J., Väyrynen J.P. (2019). Systemic inflammation in colorectal cancer: Underlying factors, effects, and prognostic significance. World J. Gastroenterol..

[19] Mármol I., Sánchez-de-Diego C., Pradilla Dieste A., Cerrada E., Rodriguez Yoldi M.J. (2017). Colorectal Carcinoma: A General Overview and Future Perspectives in Colorectal Cancer. Int. J. Mol. Sci..

[20] Floris I., Appel K., Rose T., Lejeune B. (2018). 2LARTH^®^, a micro-immunotherapy medicine, exerts anti-inflammatory effects in vitro and reduces TNF-α and IL-1β secretion. J. Inflamm. Res..

[21] Ferrà-Cañellas M.D.M., Munar-Bestard M., Garcia-Sureda L., Lejeune B., Ramis J.M., Monjo M. (2021). BMP4 micro-immunotherapy increases collagen deposition and reduces PGE2 release in human gingival fibroblasts and increases tissue viability of engineered 3D gingiva under inflammatory conditions. J. Periodontol..

[22] Lilli N.L., Révy D., Volteau C., Robelet S., Lejeune B. (2019). Effect of 2LMISEN^®^ on Long-Term Hippocampal Neurons Culture as a Screening Senescent Cells Model: p16INK4A and Caspase 3 Quantification. Adv. Aging Res..

[23] Lilli N.L., Révy D., Robelet S., Lejeune B. (2019). Effect of the micro-immunotherapy medicine 2LPARK^®^ on rat primary dopaminergic neurons after 6-OHDA injury: Oxidative stress and survival evaluation in an in vitro model of Parkinson’s disease. Degener. Neurol. Neuromuscul. Dis..

[24] Efficacy of 2LPAPI®, A Micro-Immunotherapy Drug, In Patients with High-Risk Papillomavirus Genital Infection. https://www.scirp.org/journal/paperinformation.aspx?paperid=64702.

[25] Floris I., Chenuet P., Togbe D., Volteau C., Lejeune B. (2020). Potential Role of the Micro-Immunotherapy Medicine 2LALERG in the Treatment of Pollen-Induced Allergic Inflammation. Dose-Response.

[26] Pro-Inflammatory Cytokines at Ultra-Low Dose Exert Anti-Inflammatory Effect In Vitro: A Possible Mode of Action Involving Sub-Micron Particles?. https://pubmed.ncbi.nlm.nih.gov/33633511/.

[27] Floris I., García-González V., Palomares B., Appel K., Lejeune B. (2020). The Micro-Immunotherapy Medicine 2LARTH^®^ Reduces Inflammation and Symptoms of Rheumatoid Arthritis In Vivo. Int. J. Rheumatol..

[28] Jacques C., Chatelais M., Fekir K., Fauconnier L., Mellier M., Togbe D., Floris I. (2021). The Micro-Immunotherapy Medicine 2LEID Exhibits an Immunostimulant Effect by Boosting Both Innate and Adaptive Immune Responses. Int. J. Mol. Sci..

[29] Jacques C., Chatelais M., Fekir K., Brulefert A., Floris I. (2022). The Unitary Micro-Immunotherapy Medicine Interferon-γ (4 CH) Displays Similar Immunostimulatory and Immunomodulatory Effects than Those of Biologically Active Human Interferon-γ on Various Cell Types. Int. J. Mol. Sci..

[30] Jacques C., Floris I., Lejeune B. (2021). Ultra-Low Dose Cytokines in Rheumatoid Arthritis, Three Birds with One Stone as the Rationale of the 2LARTH^®^ Micro-Immunotherapy Treatment. Int. J. Mol. Sci..

[31] Hovav A.-H. (2014). Dendritic cells of the oral mucosa. Mucosal Immunol..

[32] Wu R.-Q., Zhang D.-F., Tu E., Chen Q.-M., Chen W. (2014). The mucosal immune system in the oral cavity-an orchestra of T cell diversity. Int. J. Oral Sci..

[33] Vishnyakova P., Poltavets A., Karpulevich E., Maznina A., Vtorushina V., Mikhaleva L., Kananykhina E., Lokhonina A., Kovalchuk S., Makarov A. (2021). The response of two polar monocyte subsets to inflammation. Biomed. Pharmacother. Biomed. Pharmacother..

[34] Jiang L.N., Yao C.Y., Jin Q.L., He W.X., Li B.Q. (2011). The Enhanceing effect of IL-12 on phagocytosis and killing of Mycobacterium tuberculosis by neutrophils in tuberculosis patients. Chin. J. Cell. Mol. Immunol..

[35] Rollag H., Degré M., Sonnenfeld G. (1984). Effects of Interferon-α/β and Interferon-γ Preparations on Phagocytosis by Mouse Peritoneal Macrophages. Scand. J. Immunol..

[36] Duineveld L.A.M., van Asselt K.M., Bemelman W.A., Smits A.B., Tanis P.J., van Weert H.C.P.M., Wind J. (2016). Symptomatic and Asymptomatic Colon Cancer Recurrence: A Multicenter Cohort Study. Ann. Fam. Med..

[37] Franzè E., Laudisi F., Di Grazia A., Marônek M., Bellato V., Sica G., Monteleone G. (2020). Macrophages produce and functionally respond to interleukin-34 in colon cancer. Cell Death Discov..

[38] Mantovani A., Marchesi F., Malesci A., Laghi L., Allavena P. (2017). Tumour-associated macrophages as treatment targets in oncology. Nat. Rev. Clin. Oncol..

[39] Cortese N., Donadon M., Rigamonti A., Marchesi F. (2019). Macrophages at the crossroads of anticancer strategies. Front. Biosci. Landmark.

[40] Forssell J., Oberg A., Henriksson M.L., Stenling R., Jung A., Palmqvist R. (2007). High macrophage infiltration along the tumor front correlates with improved survival in colon cancer. Clin. Cancer Res..

[41] Park J.W., Chang H.J., Yeo H.Y., Han N., Kim B.C., Kong S.-Y., Kim J., Oh J.H. (2020). The relationships between systemic cytokine profiles and inflammatory markers in colorectal cancer and the prognostic significance of these parameters. Br. J. Cancer.

[42] Mehrotra A., D’Angelo J.A., Romney-Vanterpool A., Chu T., Bertoletti A., Janssen H.L.A., Gehring A.J. (2020). IFN-α Suppresses Myeloid Cytokine Production, Impairing IL-12 Production and the Ability to Support T-Cell Proliferation. J. Infect. Dis..

[43] Wolters T.L.C., Netea M.G., Hermus A.R.M.M., Smit J.W.A., Netea-Maier R.T. (2017). IGF1 potentiates the pro-inflammatory response in human peripheral blood mononuclear cells via MAPK. J. Mol. Endocrinol..

[44] Garg A.D., Romano E., Rufo N., Agostinis P. (2016). Immunogenic versus tolerogenic phagocytosis during anticancer therapy: Mechanisms and clinical translation. Cell Death Differ..

[45] Jin G.F., Guo Y.S., Ball C., Houston C.W. (1993). Insulin-like growth factors enhance phagocytosis by human neutrophils in vitro. Regul. Pept..

[46] Ji K.-Y., Kim K.M., Kim Y.H., Shim K.-S., Lee J.Y., Kim T., Chae S. (2021). Serum Starvation Sensitizes Anticancer Effect of Anemarrhena asphodeloides via p38/JNK-Induced Cell Cycle Arrest and Apoptosis in Colorectal Cancer Cells. Am. J. Chin. Med..

[47] Yao G. (2014). Modelling mammalian cellular quiescence. Interface Focus.

[48] Hanahan D., Weinberg R.A. (2011). Hallmarks of Cancer: The Next Generation. Cell.

[49] Koveitypour Z., Panahi F., Vakilian M., Peymani M., Seyed Forootan F., Nasr Esfahani M.H., Ghaedi K. (2019). Signaling pathways involved in colorectal cancer progression. Cell Biosci..

[50] Spano J.P., Fagard R., Soria J.-C., Rixe O., Khayat D., Milano G. (2005). Epidermal growth factor receptor signaling in colorectal cancer: Preclinical data and therapeutic perspectives. Ann. Oncol..

[51] Mitsudomi T., Yatabe Y. (2010). Epidermal growth factor receptor in relation to tumor development: EGFR gene and cancer. FEBS J..

[52] Cho Y.B., Hong H.K., Choi Y.-L., Oh E., Joo K.M., Jin J., Nam D.-H., Ko Y.-H., Lee W.Y. (2014). Colorectal cancer patient-derived xenografted tumors maintain characteristic features of the original tumors. J. Surg. Res..

[53] Engstrand J., Nilsson H., Strömberg C., Jonas E., Freedman J. (2018). Colorectal cancer liver metastases—A population-based study on incidence, management and survival. BMC Cancer.

[54] Huot J.R., Novinger L.J., Pin F., Bonetto A. (2020). HCT116 colorectal liver metastases exacerbate muscle wasting in a mouse model for the study of colorectal cancer cachexia. Dis. Model Mech..

[55] Fujimoto K., Fujii G., Sakurai H., Yoshitome H., Mutoh M., Wada M. (2015). Intestinal Peyer’s patches prevent tumorigenesis in ApcMin/+ mice. J. Clin. Biochem. Nutr..

[56] Tseng W., Leong X., Engleman E. (2007). Orthotopic Mouse Model of Colorectal Cancer. J. Vis. Exp..

[57] Szadvari I., Krizanova O., Babula P. (2016). Athymic nude mice as an experimental model for cancer treatment. Physiol. Res..

[58] Budzynski W., Radzikowski C. (1994). Cytotoxic cells in immunodeficient athymic mice. Immunopharmacol. Immunotoxicol..

[59] De Jong M., Essers J., van Weerden W.M. (2014). Imaging preclinical tumour models: Improving translational power. Nat. Rev. Cancer.

[60] Tachibana T., Oyama T.G., Yoshii Y., Hihara F., Igarashi C., Tsuji A.B., Higashi T., Taguchi M. (2021). Establishment of an In Vivo Xenograft Mouse Model of a Subcutaneous Submillimeter HT-29 Tumor Formed from a Single Spheroid Transplanted Using Radiation-Crosslinked Gelatin Hydrogel Microwell. Appl. Sci..

[61] Atzpodien J., Kirchner H., Hänninen E.L., Deckert M., Fenner M., Poliwoda H. (1993). Interleukin-2 in combination with interferon-alpha and 5-fluorouracil for metastatic renal cell cancer. Eur. J. Cancer.

[62] Mirlekar B., Pylayeva-Gupta Y. (2021). IL-12 Family Cytokines in Cancer and Immunotherapy. Cancers.

[63] Colombo M.P., Trinchieri G. (2002). Interleukin-12 in anti-tumor immunity and immunotherapy. Cytokine Growth Factor Rev..

[64] Zaharoff D.A., Hance K.W., Rogers C.J., Schlom J., Greiner J.W. (2010). Intratumoral immunotherapy of established solid tumors with chitosan/IL-12. J. Immunother..

[65] Coca S., Enrech S., Moreno García V., Sáez M.A., Gutiérrez C., Colmenarejo A., Hernández J.M., Pérez Piqueras J. (2005). Evaluation of the antitumor activity of interleukin-12 in an experimental murine model of colorectal cancer induced by 1,2 dimethyl-hydrazine (DMH). Rev. Esp. Enferm. Dig..

[66] Hande K.R. (1998). Etoposide: Four decades of development of a topoisomerase II inhibitor. Eur. J. Cancer.

[67] Burden D.A., Kingma P.S., Froelich-Ammon S.J., Bjornsti M.-A., Patchan M.W., Thompson R.B., Osheroff N. (1996). Topoisomerase II·Etoposide Interactions Direct the Formation of Drug-induced Enzyme-DNA Cleavage Complexes. J. Biol. Chem..

[68] Kang K., Oh S.H., Yun J.H., Jho E.H., Kang J.-H., Batsuren D., Tunsag J., Park K.H., Kim M., Nho C.W. (2011). A Novel Topoisomerase Inhibitor, Daurinol, Suppresses Growth of HCT116 Cells with Low Hematological Toxicity Compared to Etoposide. Neoplasia.

[69] Heiduschka G., Bigenzahn J., Brunner M., Thurnher D. (2014). Resveratrol synergistically enhances the effect of etoposide in HNSCC cell lines. Acta Otolaryngol..

[70] Vernousfaderani E.K., Akhtari N., Rezaei S., Rezaee Y., Shiranirad S., Mashhadi M., Hashemi A., Khankandi H.P., Behzad S. (2021). Resveratrol and Colorectal Cancer: A Molecular Approach to Clinical Researches. Curr. Top. Med. Chem..

[71] Athar M., Back J.H., Kopelovich L., Bickers D.R., Kim A.L. (2009). Multiple molecular targets of resveratrol: Anti-carcinogenic mechanisms. Arch. Biochem. Biophys..

[72] Schwager J., Richard N., Widmer F., Raederstorff D. (2017). Resveratrol distinctively modulates the inflammatory profiles of immune and endothelial cells. BMC Complement. Altern. Med..

[73] Buttari B., Profumo E., Segoni L., D’Arcangelo D., Rossi S., Facchiano F., Saso L., Businaro R., Iuliano L., Riganò R. (2014). Resveratrol Counteracts Inflammation in Human M1 and M2 Macrophages upon Challenge with 7-Oxo-Cholesterol: Potential Therapeutic Implications in Atherosclerosis. Oxid. Med. Cell. Longev..

[74] Sheng J., Sun H., Yu F.-B., Li B., Zhang Y., Zhu Y.-T. (2020). The Role of Cyclooxygenase-2 in Colorectal Cancer. Int. J. Med. Sci..

[75] Szewczuk L.M., Forti L., Stivala L.A., Penning T.M. (2004). Resveratrol is a peroxidase-mediated inactivator of COX-1 but not COX-2: A mechanistic approach to the design of COX-1 selective agents. J. Biol. Chem..

[76] Salla M., Pandya V., Bhullar K.S., Kerek E., Wong Y.F., Losch R., Ou J., Aldawsari F.S., Velazquez-Martinez C., Thiesen A. (2020). Resveratrol and Resveratrol-Aspirin Hybrid Compounds as Potent Intestinal Anti-Inflammatory and Anti-Tumor Drugs. Molecules.

[77] Shaito A., Posadino A.M., Younes N., Hasan H., Halabi S., Alhababi D., Al-Mohannadi A., Abdel-Rahman W.M., Eid A.H., Nasrallah G.K. (2020). Potential Adverse Effects of Resveratrol: A Literature Review. Int. J. Mol. Sci..

[78] Berardi V., Ricci F., Castelli M., Galati G., Risuleo G. (2009). Resveratrol exhibits a strong cytotoxic activity in cultured cells and has an antiviral action against polyomavirus: Potential clinical use. J. Exp. Clin. Cancer Res..

[79] Kornienko J.S., Smirnova I.S., Pugovkina N.A., Ivanova J.S., Shilina M.A., Grinchuk T.M., Shatrova A.N., Aksenov N.D., Zenin V.V., Nikolsky N.N. (2019). High doses of synthetic antioxidants induce premature senescence in cultivated mesenchymal stem cells. Sci. Rep..

[80] Koch J., Mönch D., Maaß A., Gromoll C., Hehr T., Leibold T., Schlitt H.J., Dahlke M.-H., Renner P. (2021). Three dimensional cultivation increases chemo- and radioresistance of colorectal cancer cell lines. PLoS ONE.

[81] Obinu A., Rassu G., Corona P., Maestri M., Riva F., Miele D., Giunchedi P., Gavini E. (2019). Poly (ethyl 2-cyanoacrylate) nanoparticles (PECA-NPs) as possible agents in tumor treatment. Colloids Surf. B Biointerfaces.

[82] Fiorentino F.P., Marchesi I., Schröder C., Schmidt R., Yokota J., Bagella L. (2020). BET-Inhibitor I-BET762 and PARP-Inhibitor Talazoparib Synergy in Small Cell Lung Cancer Cells. Int. J. Mol. Sci..

