# Supplementary material for: A Micro-Immunotherapy Sequential Medicine MIM-seq Displays Immunomodulatory Effects on Human Macrophages and Anti-Tumor Properties towards In Vitro 2D and 3D Models of Colon Carcinoma and in an In Vivo Subcutaneous Xenograft Colon Carcinoma Model
Source: Int J Mol Sci. 2022 May 27;23(11):6059. doi: 10.3390/ijms23116059 (PMC9181410; doi:10.3390/ijms23116059)
Supplement: Supplementary file 1 [file ijms-23-06059-s001.zip › ijms-1727134-supplementary.pdf]

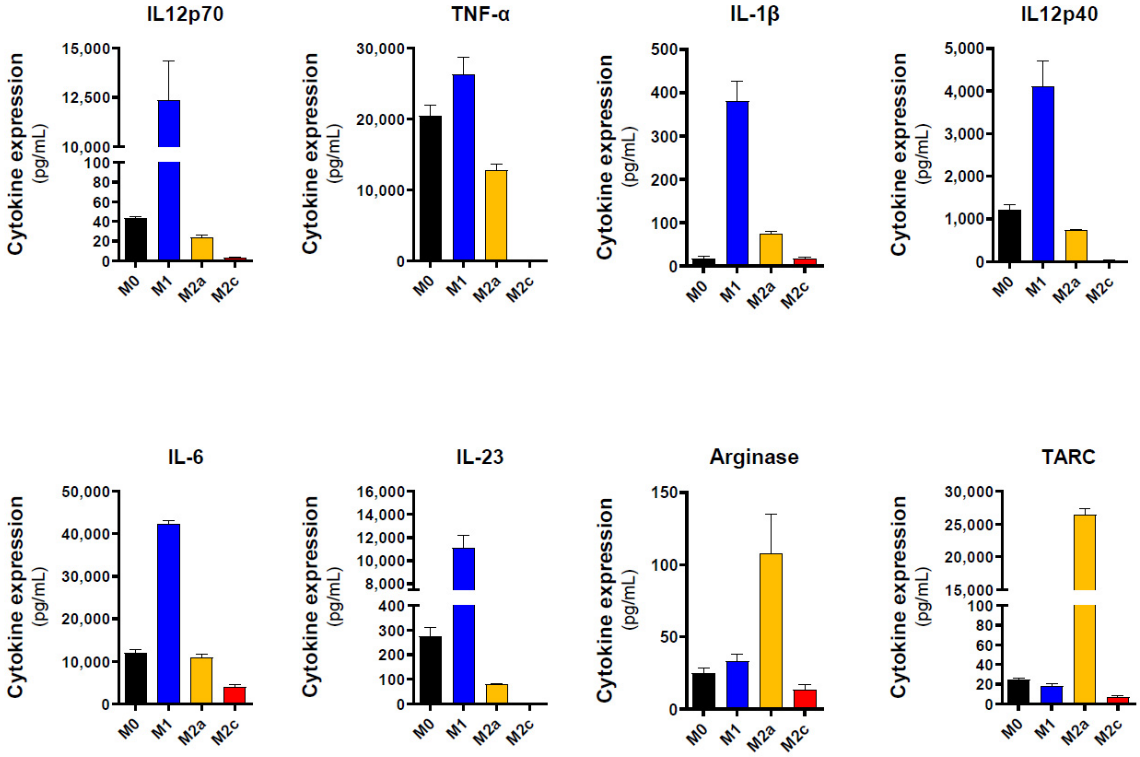

Supplementary Figure S1: Evaluation of the cytokine secretion within the culture supernatant, in control conditions, for the different macrophage's subpopulations. The secretion of IL12p70, TNF-α, IL-1β, IL12p40, IL-6, IL-23, arginase and TARC (expressed in pg/mL) was assessed by ELISA assay after macrophage polarization in presence of either 20 ng/mL IFN-γ (M1; blue bars), 5 ng/mL IL-4 (M2a; yellow bars), or 20 ng/mL IL-10 (M2c; red bars). The M0 unpolarized population (black bars) is also represented. In this setting, the macrophage polarization process is a seven-day experiment, where the cells are incubated in presence of the above-mentioned differentiation cytokines. In the last 24 hours of incubation, all the cells' subpopulations were stimulated with LPS (100 ng/mL), as an inducer of the cytokine secretion.

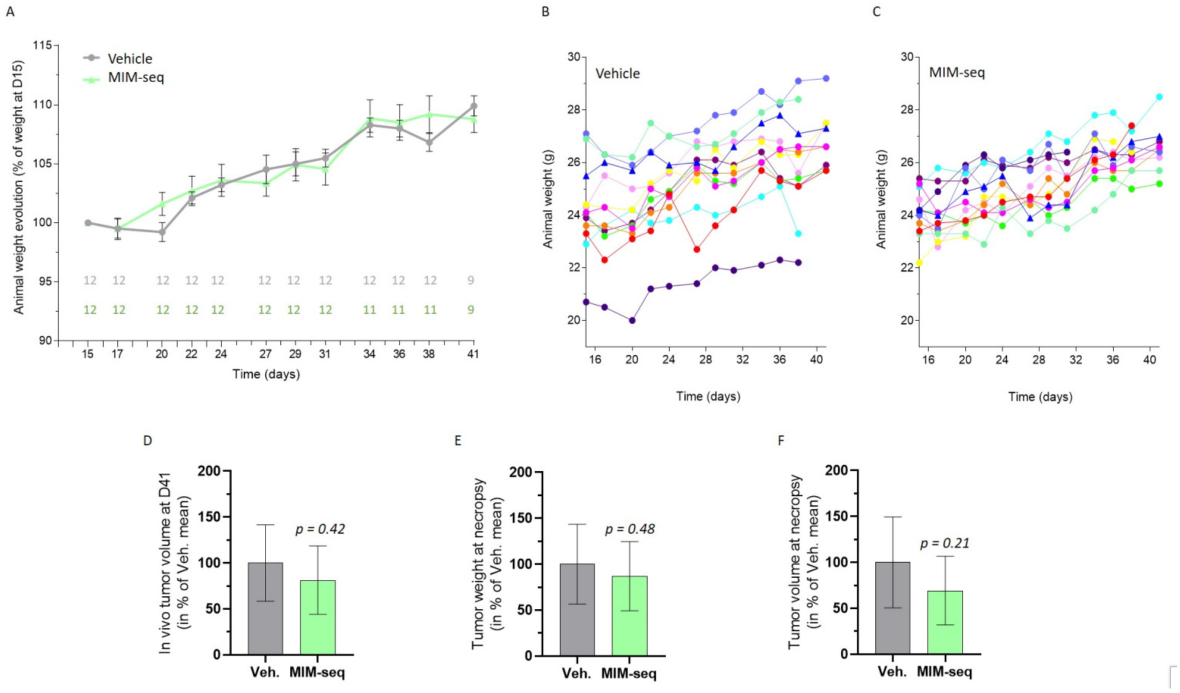

Supplementary Figure S2: MIM-seq effects on body weight, in vivo tumor volume at D41 and tumor weight and volume at the time of necropsy. (**A-C**) Animal weights evolution. After randomization on Day 15 (D15), vehicle and MIM-seq were administered by daily oral gavage until Day 41 (D41). Animal weights were measured three times weekly. Resulting animal weights are displayed as mean ±SD evolution of animal weights in percentages of weight at D15 and are represented with points and connecting lines with standard deviation bars (the number of mice at each time point is reported in the graph, in grey for the Veh. group and in green for the MIM-seq group). Resulting individual animal weights (**B-C**) in the Veh. - and in the MIM-seq-treated groups are displayed with points and connecting lines and expressed in grams (initial n at D15 = 12 mice in each group). Each color represents the weight evolution for one mouse per group. (**D**) The in vivo tumor volume at D41 is represented in percentage of vehicle mean ±SD (p = 0.42). (**E**) The tumor weight at necropsy (p = 0.48) and (**F**) the tumor volume at necropsy (p = 0.21), expressed in percentage of vehicle mean ±SD are represented. For the three latter representations, the two groups are compared with Wilcoxon-Mann-Whitney tests.

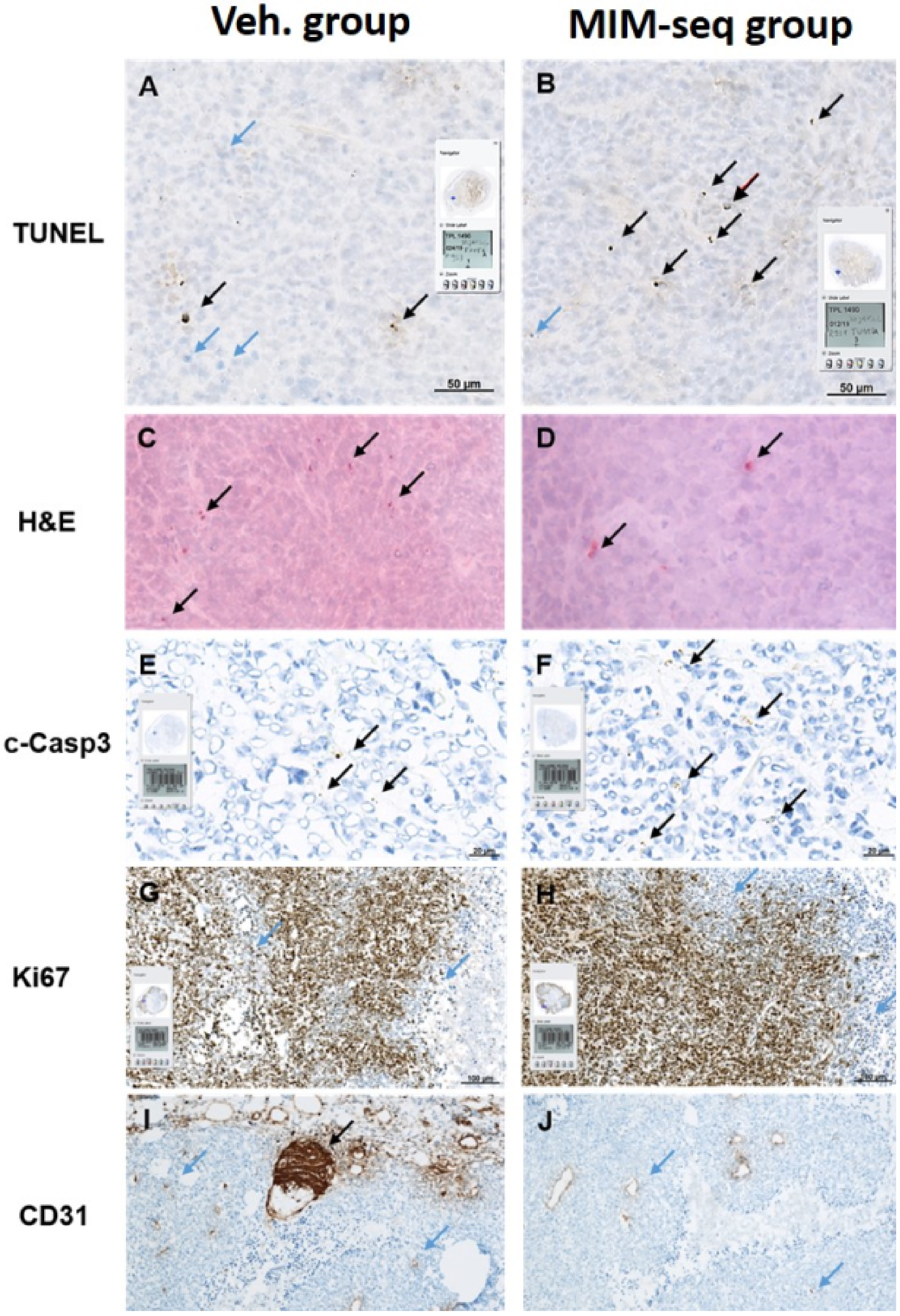
Veh. group
MIM-seq group
A
B
TUNEL
50 µm
50 µm
C
D
H&E
E
F
c-Casp3
20 µm
20 µm
G
H
Ki67
I
J
CD31

Supplementary Figure S3: Representative pictures of tumor tissue at high or medium magnification, stained with TUNEL Assay (**A, B**), routine H&E (**C, D**), cleaved caspase 3 (**E, F**), Ki67 (**G, H**) and CD31 (**I, J**) within the Veh.-treated group and the MIM-seq-treated group.

A and B: The density of the TUNEL+ cells undergoing apoptosis is generally low to very low. In (**A**) Veh.-treated tumors, the TUNEL+ cells are often slightly sparser than those present in (**B**) MIM-seq-treated tumors (black arrows), whereas the TUNEL negative cells in individual cell necrosis with condensed nuclei are relatively more numerous (blue arrows).

C and D: The density of cells exhibiting individual cell death (condensed cells with condensed of smudgy nuclei and/or hypereosinophilic cytoplasm) is generally slightly higher in Veh.-treated tumors than in MIM-seq-treated tumors (black arrows).

E and F: Numbers of c-Casp3 positive cells are generally minimal. There is no perceptible difference of c-Casp3+ cells density comparing Veh.-treated (**E**) and MIM-seq-treated (**F**) tumors (black arrows).

G and H: Nearly all intact cells are Ki67 positive, with abrupt arrest of cell cycling within and immediately adjacent to areas undergoing ischemic necrosis (blue arrows) with no substantial difference between the 2 treatment groups.

I and J: Peripheral deeply CD31+ platelet thrombi were noted at tumor periphery among the intact neoplastic tissue in 2/6 Veh.-treated mice (black arrow in **I**), whereas none were present in the 6 MIM-seq-treated mice (**J**). Vascular density was otherwise similar, comparing the Veh. control and MIM-seq-treated groups (blue arrows). All molecular markers were labelled with diaminobenzidine (DAB) and counterstained with light Meyer's hematoxylin with bluing reagents.
